# Blended and Microparticle Composite Hyaluronan Hydrogels with Programmable Degradation through Selective Oxidation

**DOI:** 10.1021/acspolymersau.5c00129

**Published:** 2025-12-17

**Authors:** Melanie Grimm, Fiona Ye Rojo Acero, Fatemeh Safari, Desiré Venegas-Bustos, Andreas Wagner, Clara Presciutti, Wen Chen, Matteo D’Este, Jacek K. Wychowaniec

**Affiliations:** † 161930 AO Research Institute Davos, Clavadelerstrasse 8, Davos 7270, Switzerland; ‡ ETH Zürich, Rämistrasse 101, Zürich 8092, Switzerland; § Bioforge Lab, LaDIS, CIBER-BBN, Edificio LUCIA, 16782 Universidad de Valladolid, Valladolid 47011, Spain; ∥ 272858 Anton Paar GmbH, Anton-Paar-Str. 20, 8054 Graz, Austria

**Keywords:** tyramine-modified hyaluronan, oxidation, molecular weight, degradation, hydrogels

## Abstract

The design space of hydrogels for biomedical applications embraces a wide variety of parameters that can be tuned through chemical modification. Among them, tissue adhesion and viscoelastic properties contribute to the integration of tissue-engineered constructs with native tissues, while the degradation profile determines their temporal evolution and cell invasion. Selective 1,2-diol oxidation is a versatile tool to control all of these properties in polysaccharide-based hydrogels by generating aldehyde groups. A key challenge in implementing this tool is that although aldehyde groups improved adhesion, they also promoted chain fragmentation, demanding a trade-off. To address this, we devised a strategy that leverages the adhesiveness of oxidized biopolymers together with the mechanical stability of their nonoxidized counterparts. Here, we synthesized tyramine-modified hyaluronan (THA) and its oxidized form (oTHA) and evaluated their degradation and adhesion in various combinations and formats, including *blended hydrogels* and *hydrogel microparticle composite* networks. As the degree of oxidation increased in oTHA, its molecular weight decreased, the storage modulus of the resulting hydrogels slightly declined, brittleness increased, and physical degradation accelerated. These opposing properties were finely offset in two-component blended hydrogels; increasing the oTHA content proportionally accelerated the degradation rate in both bulk and hydrogel microparticle composite formats while maintaining consistent viscoelastic properties and network topology at a fixed total polymer concentration. By adjusting the oTHA-to-THA ratio, we generated composite hydrogels with two distinct degradation behaviors: (i) *collapse-type mode*, where *blended hydrogels* gradually softened and spread without fragmenting; and (ii) *fragmentation-type mode*, where *hydrogel microparticle composites* abruptly broke into discrete pieces over degradation time. This tunability enables the design of a new class of composite soft biomaterials with programmable degradation. Such materials show potential for tunable tissue engineering strategies, which could be implemented for controlling cell invasion, migration, and proliferation in biological applications.

## Graphical abstract

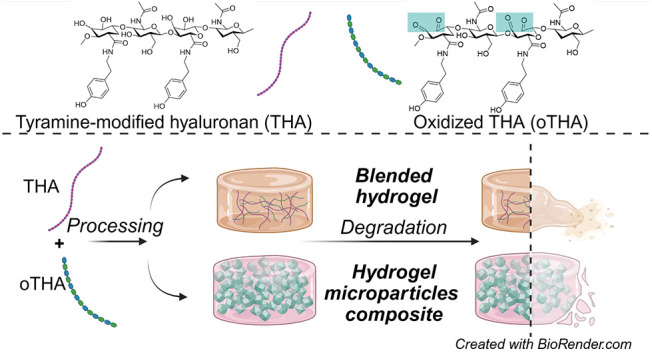

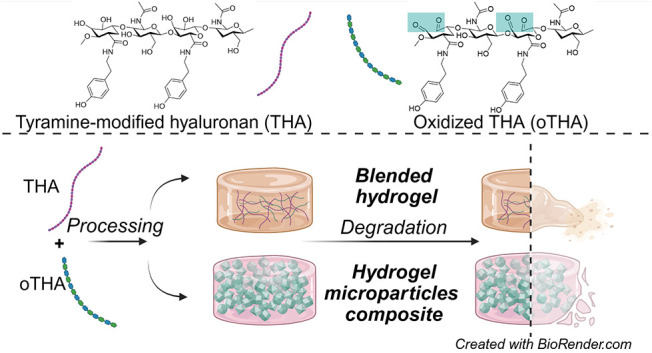

## 1 Introduction

Despite recent advances in the development of three-dimensional (3D) printable and implantable biomaterials in the field of musculoskeletal tissue engineering, there is still a clear need for versatile materials for regenerative and therapeutic applications. Implanted biomaterials available in the field could be improved by simultaneous (i) appropriate adhesion to surrounding host tissues *in vivo*, (ii) controlled degradation matching the desired tissue regeneration outcome, and (iii) enabling controlled cellular invasion from neighboring tissues to allow tissue replacement.^1^ Regarding functional inks for 3D printing, the main complexity in the their development is that the materials, typically biopolymeric hydrogels, need a narrow and somewhat opposite set of physical and biological properties, enabling degradation, adhesion, as well as cellular invasion, while maintaining accurate viscoelastic properties.^2,3^

Hydrogels are widely used in tissue engineering due to their similarity to the extracellular matrix, water retention capabilities, and potential for chemical modification, and are prone to composite biomaterial formation.^4–7^ Moreover, engineering complex biomimetic constructs to support cell function for tissue engineering that recapitulate the native environment requires (i) rheological properties suitable for fabrication/structuring,^8–10^ (ii) tunable stiffness to influence cell fate/lineage,^11,12^ and (iii) the ability to solidify and remain intact for extended periods in complex aqueous environments, while having controlled degradation profiles,^13–16^ many of which can be fulfilled by hydrogels. For an advanced cell support, a careful balance between adhesion, degradation, and viscoelastic properties is therefore required.^17–20^ These factors are determined by the polymer molecular weight (*M*_w_), concentration, and charge, and by the extent of entanglement and network topology, which are influenced by intermolecular interactions.^21–25^

Multicomponent polymer (*e.g*., blended) networks offer the possibility of tuning these interactions by adjusting their composition. To date, a large number of multicomponent hydrogel-based formulations have been tested for biofabrication, including mixtures of ionic cross-linked alginate, temperature cross-linked agarose and collagen, as well as chemically modified hyaluronan (HA) or gelatin biopolymers.^1,8,10,26^ The main challenge with these systems is to optimize the formulation from the initial stability in the polymer solution to the formation of a stable and accurate cytocompatible 3D structure that can adhere to the host tissue, degrade on demand to match the local environmental remodeling, and instruct local neighboring cells to invade its structure for this remodeling to occur. Another challenge is that different fabrication steps require vastly different rheological properties, making all these properties highly interdependent. Hydrogels composed of two independently cross-linked components or multiple chemistries have been proposed to tackle these issues.^1^ Typically, by combining at least two polymeric precursors that can undergo gelation through two independent mechanisms, inks can be produced with rheological, adhesiveness, and degradability features adjusted via the cross-linking of one or the other component before and after injection/extrusion, as well as before and after *in vivo* implantation.^1^

In recent years, granular hydrogels and hydrogel microparticle composites with particle-based architectures have emerged as alternatives to conventional bulk hydrogels.^27^ Their modular, particle-based architectures often enable intrinsic high injectability due to shear-thinning behavior, with rapid post injection self-stabilization without the need for additional cross-linking methods.^28^ Moreover, the interstitial voids between these materials may provide intrinsic microporosity that enhances nutrient diffusion, promotes cellular infiltration, and supports tissue integration. In hydrogel microparticle composites, the combination of a continuous hydrogel phase with discrete microparticles introduces multiscale mechanical and biological tunability, allowing for the possible decoupling of stiffness and therapeutic function within a single system.

HA is a natural glycosaminoglycan and a negatively charged polysaccharide with a linear structure. It is composed of alternating dimeric (1 → 4)-β-linked d-glucuronic and (1 → 3)-β-linked *N*-acetyl-d-glucosamine residues. HA plays a key biological role in a range of human tissues; however, its solutions in physiological or biological media are characterized by rapid degradation and poor mechanical properties.^29,30^ It is known that the biological effects of HA depend on its *M*_w_,^31–33^ which is a polymer property that defines the overall stability of the formed hydrogels in complex physiological settings. HA-based hydrogels have been widely used in tissue engineering and regenerative medicine due to their biocompatibility, tunable physicochemical properties, and potential for in situ cross-linking.^34–39^ To enhance the stability of HA and enable the development of injectable and stimuli-responsive hydrogel systems, a range of chemical strategies have been employed.^1,40–47^ For instance, the addition of aldehyde groups to modify HA (into oxidized oHA) improves cross-linking with various amine-bearing polymers such as gelatin or carboxymethyl chitosan via Schiff-base reactions, allowing rapid gelation under physiological conditions without external stimuli.^41,48,49^ Selective oxidation introduces aldehyde groups capable of forming covalent bonds with amines under physiological conditions, thereby enabling spontaneous adhesion of the hydrogel to biological tissues.^1^ An oxidation degree of around ∼8% in oTHA may provide sufficient adhesiveness to host tissues, while ensuring the degradation rate that matches the rate of tissue formation *in vivo.* ^1^ However, these systems rapidly degrade via hydrolysis or in the presence of hyaluronidase, with degradation times ranging from 24 h to 4 days (in 100 U mL^–1^ hyaluronidase), depending on the polymer *M*_w_ and oxidation degree.^41,48,49^ To address this limitation, Jia et al. explored multiple modification strategies, including methacrylation (HAMA), partial oxidation followed by methacrylation (oHAMA), and synthetic poly­(*N*,*N*-dimethylacrylamide) (P­(DMAM)) copolymer grafting prior to oxidation and methacrylation (oHA-*g*-P)/MA, and reported degradation times ranging from several days to weeks in phosphate buffer saline (PBS) and hyaluronidase (5 U mL^–1^).^44^ Although these approaches broaden the tunable degradation window, the reliance on extensive chemical modification and synthetic copolymer incorporation may complicate biocompatibility and translational potential compared to simpler HA-only systems.

A key challenge in designing adaptable systems, however, still lies in the trade-off introduced by oxidation: although the addition of aldehyde groups enhances adhesion, it also reduces *M*_w_ and promotes chain fragmentation.^44,48,50–52^ This chain fragmentation in periodate oxidation occurs via cleavage of the vicinal diols on the glucuronic acid and N-acetylglucosamine residues, resulting in the formation of reactive aldehyde groups through ring opening of the sugar units.^51,53,54^ This oxidative cleavage may also break the glycosidic backbone at the C2–C3 position,^53^ leading to depolymerization and a significant reduction in the *M*_w_. As a result, the physical stability of the formed hydrogels is reduced, and more pro-inflammatory fragments may be formed.^55,56^ To address this issue, in this work, we propose a new strategy that potentially leverages the adhesiveness of oxidized oHA while maintaining the mechanical stability of its nonoxidized counterpart. To achieve this, we synthesized tyramine-modified hyaluronan (THA) and its oxidized form (oTHA, Figure 1A) and evaluated their mechanical properties, degradation, and adhesion in various combinations and formats, including (i) single-component bulk hydrogels, *i.e*., either THA or oTHA; (ii) blended hydrogels consisting of both oTHA and THA (named hereafter as *two-component blended hydrogels*, Figure 1B), as well as (iii) oTHA microgels (oTHAm) embedded in THA networks (named hereafter as *hydrogel microparticle composite(s)*, Figure 1C). In this study, a *hydrogel microparticle composite* approach was selected to extend the possible tunability achieved in two-component THA/oTHA *blended hydrogels* by introducing the possibility to spatially control degradation via changes in the underlying network topology. The changes in the architecture may enable decoupling of adhesion and degradation behavior, offering a new perspective for enhanced control over network disintegration and mechanical integrity compared to previously demonstrated homogeneous bulk formulations in this field.^57–60^

**Figure 1:**
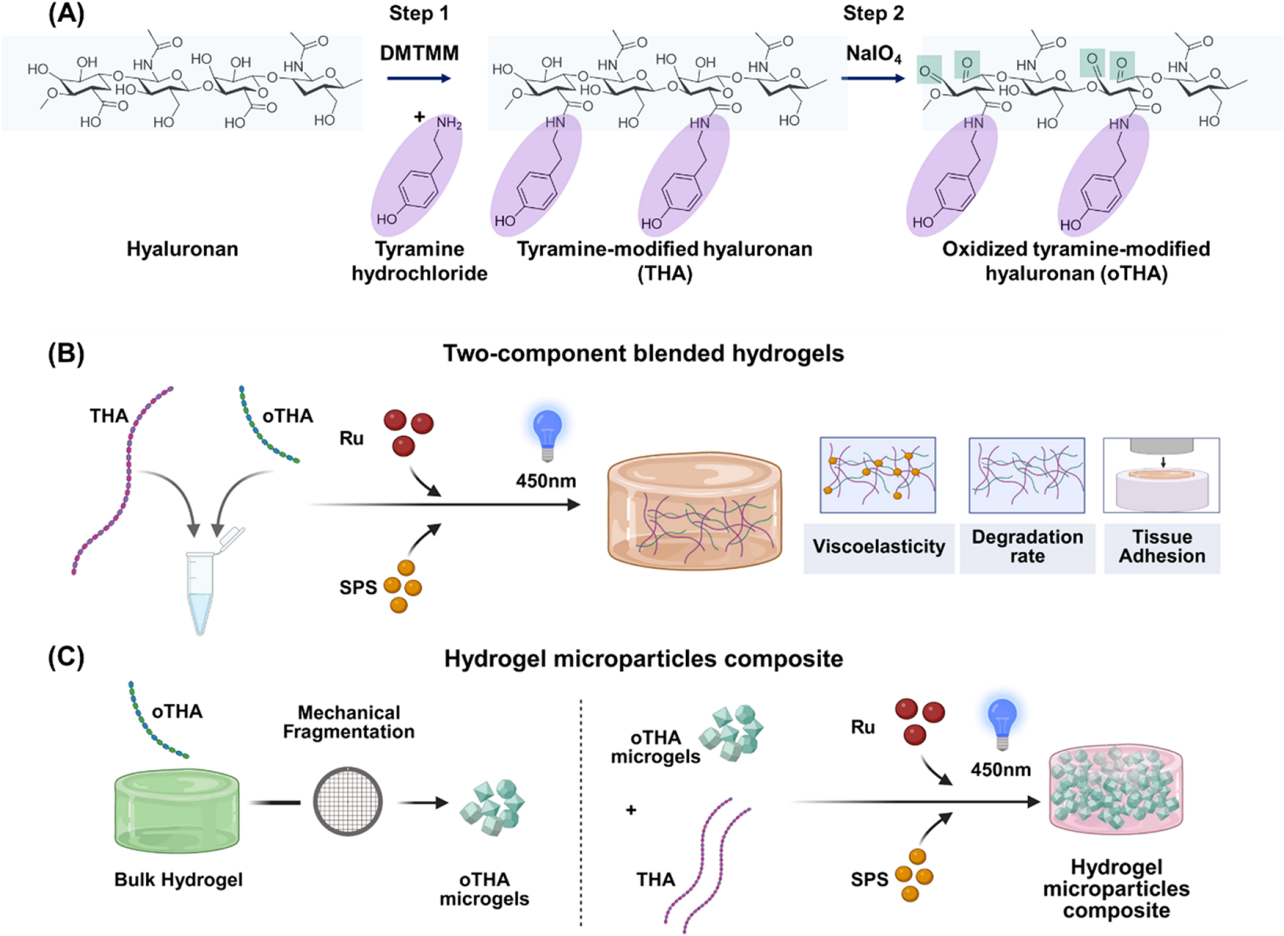
Schematic approaches for the generation of tunable hydrogels consisting of modified hyaluronic acid. (A) Reactions of tyramine-modified hyaluronan (step 1, THA) and oxidized tyramine-modified hyaluronan (step 2, oTHA). (B) Mixing of oTHA with THA and the formation of two-component blended hydrogels approach, as well as the ways in which the main properties (viscoelasticity, degradation rate, and adhesion) can be controlled by the design. (C) Generation of hydrogel microparticle composites. First bulk oTHA hydrogels were formulated and mechanically fragmented into microgels. These were then incorporated into an interstitial THA matrix to generate hydrogel microparticle composites. Schemes (B, C) were created with BioRender.com.

## 2 Results and Discussion

### 2.1 Oxidation of THA and Its Effects on the Molecular Weight

HA with *M*_w_ = 280–290 kDa (abbreviated as LMW) and HA with *M*_w_ = 1.64 MDa (abbreviated as HMW) were used as precursors for the subsequent syntheses (Figure 1A, Table 1, Section 4). We first synthesized several LMW and HMW THA batches with an average degree of tyramine substitution (DoS) across all of them of 5.72 ± 0.26%. The DoS of tyramine substitution was kept constant across all the batches used for the subsequent preparation of oTHA. To produce hydrogels with variable degrees of oxidation, we readapted the synthesis of oTHA from previous work,^1,61^ and chose three molar ratios of NaIO_4_ to THA, namely, 1:1, 2:3, and 1:3, aiming at **high**, **mid**, and **low** degrees of oxidation (DoO) (Table 1).^1^ To assess robustness and reproducibility, we ran periodate oxidation using independent sets of reactions, as detailed in Table 1. To minimize variability, all syntheses within each set were run in parallel, under the same conditions, with only the ratio of NaIO_4_ to THA changing (Table 2 and Section 4.2 in Materials and Methods). All synthesized oTHA batches were first characterized for their DoO using the Purpald reagent calorimetric test, which showed the expected trend and consistency across all sets (Table 1). The ^1^H NMR data of all sets A–C of the used THA and oTHA batches are presented in Figures S1–S3, respectively. Minor differences were noted in the 4.5–5.5 ppm region for the oTHA samples compared to THA, specifically the appearance of the peaks at 4.9, 5.0, and 5.12 ppm. These signals were attributed to local environmental changes in the hyaluronan structure resulting from aldehyde formation upon oxidation, as also reported previously.^41^ The successful reaction was further confirmed by Fourier-transform infrared (FTIR) spectroscopy, where the presence of aldehyde groups for oTHA samples was detected with a characteristic shoulder peak at 1732 ± 2 cm^–1^ (Figure S4), corresponding to the C=O stretching vibration of aldehydes.^1^ This was however absent in their nonoxidized counterparts, as evidenced by the calculated ratios of the aldehyde group to the four key vibrational modes persisting across the FTIR spectrum of our modified oTHA samples, namely, aldehyde versus C–O–C stretching (*I*_1732_/*I*_1610_), aldehyde versus amide II (*I*_1732_/*I*_1557_), aldehyde versus C–H bending (*I*_1732_/*I*_1370_), and aldehyde versus C–O stretching (*I*_1732_/*I*_1030_).^62^ Indeed, we observed a significant increase of all four ratios for all oTHA samples, as compared to the nonoxidized THA, further confirming the successful oxidation reaction (Table S1).

**Table 1:** Degree of Oxidation (DoO) Obtained for All Independently Synthesized Batches of oTHA Prepared in Three Sets of Syntheses (A, B, and C)

			set A	set B	set C
sample	molar ratio of NaIO_4_:THA	degree of oxidation	DoO [%]		
	From LMW THA (synthesized from original HA with *M*_w_ = 280–290 kDa)				
oTHA1	1:1	high	18.1 ± 2.4	13.5 ± 2.4	17.5 ± 1.9
oTHA2	2:3	mid	11.4 ± 2.1	10.1 ± 1.1	10.1 ± 2.3
oTHA3	1:3	low	5.1 ± 0.4	3.6 ± 0.3	6.0 ± 0.8
	From HMW THA (synthesized from original HA with *M*_w_ = 1.64 MDa)				
oTHA4	1:1	high	19.2 ± 3.1	15.1 ± 1.0	-
oTHA5	2:3	mid	13.7 ± 1.3	11.1 ± 1.6	-
oTHA6	1:3	low	6.6 ± 0.8	4.7 ± 1.3	-

**Table 2:** Reactant Amounts Used for **Set A** and **Set B** of oTHA Synthesis and the Amount of Ethylene Glycol Used to Quench the Reactions

sample	THA [g]	mili-Q H_2_O [mL]	90 mg mL^**–1**^ NaIO_4_ [mL]	molar ratio of NaIO_4_:THA	ethylene glycol [mL]
From LMW THA (synthesized from original HA with *M*_w_ = 280–290 kDa)					
oTHA1	1	100	11.68	1:1	6
oTHA2	1	100	7.79	2:3	4
oTHA3	1	100	3.89	1:3	2
From HMW THA (synthesized from original HA with *M*_w_ = 1.64 MDa)					
oTHA4	1	100	11.68	1:1	6
oTHA5	1	100	7.79	2:3	4
oTHA6	1	100	3.89	1:3	2

Next, to confirm the physical changes in the behavior of the synthesized oTHAs, we performed flow viscosity measurements on 2 w/v% solutions of the synthesized polymers in PBS (Figure S5). Across all studied batches, as the DoO increased, a significant decrease of the viscosity (at a shear rate of γ̇ = 10 s^–1^), as compared to nonoxidized THA, was observed (Figure S5). Stepwise differences between individual oTHA as a function of DoO were observed; however, they were minimal and not statistically significant. This sudden decrease in viscosity was indicative of the successful oxidation reaction, consequent chain cleavage, and assumed decrease in *M*_w_. The similar values obtained across the sets of oTHA1–3 and oTHA4–6, synthesized from LMW and HMW THA, nevertheless, suggested that the oxidation reaction led to similar *M*_w_ across all syntheses, indicating that the decrease was more pronounced when starting from HA with a nominal *M*_w_ of 1.64 MDa. On average, the viscosity decreased 8-fold for LMW THA → oTHA1–3 and 28-fold for HHMW THA → oTHA4–6, indicating notable chain fragmentation. Longer molecular chains present more potential scission sites, with a consequent higher statistical likelihood of fragmentation. Additionally, breaking of HMW chains drastically reduces their entanglements and weight-average molecular weight (*M*_w_), with more visible consequences on their physicochemical properties. SEC-MALS analysis confirmed significant chain fragmentation during tyramination, particularly for the HMW THA sample, with up to ∼75% reduction in *M*_w_ relative to native HA (Table S2). At the same time, SEC-MALS showed that the average *M*_w_ of oTHA1–3 synthesized from LMW HA was reduced to 18–28% of the nominal value, whereas oTHA4–6 synthesized from HWM HA was reduced to barely 1–2% of the nominal value. Static light scattering (SLS) and dynamic light scattering (DLS) measurements supported these findings for unmodified and THA samples, but oTHA samples exhibited strong aggregation across the tested conditions, preventing reliable molecular mass determination. Full experimental details and data are provided in Section S2 of the Supporting Information (Table S2 and Figures S6–S12). Overall, our data suggest that oxidative cleavage during periodate oxidation leads to a more pronounced depolymerization when the synthesis is carried out with HMW HA, limiting the practical advantage of using HMW HA as a starting material.

### 2.2 Comparison of THA Versus oTHA Single-Component Hydrogels

Next, we compared the behavior of single-component hydrogels made of THA and oTHA. Ruthenium (Ru) and sodium persulfate (SPS) enable tunable photocross-linking of tyramine-modified polymers.^63–65^ Miklosic et al. showed that SPS governs the viscoelasticity of THA bioinks,^63^ so we fixed Ru to 0.1 mM to slow gelation in ambient conditions and set SPS at 5 mM to provide sufficient radical generation to reach higher storage modulus values. These were fixed across all formulations prepared at 2 w/v% of THA or oTHA to compare the effects of oxidation and aldehyde content on the overall changes in the viscoelastic properties of the formed hydrogels.

In photorheology, all hydrogels were cross-linked upon switching the light and reached their maximum storage modulus values within 30 s (Figures S13 and S14). The maximum storage modulus values are presented in Figures 2A and S15. The results showed that more oxidized oTHAs are characterized by a lower storage modulus. This is likely due to the fragmentation of polymer chains and reduced *M*_w_, which impacts the overall cross-linking. For an ideal entangled polymer system, *M*_w_ may be used to rationalize the viscoelastic behavior in the vicinity of the rubbery plateau via entanglement scaling, where the plateau modulus follows $G_{N}^{0}∼\frac{\rho \mathrm{RT}}{M_{e}}$ , which holds true only for a limited number of polymers.^66^ In contrast, in chemically cross-linked hydrogels like ours, no direct correlation with *M*_w_ exists since their viscoelastic response is intrinsically dictated by the cross-linking density, connectivity, and network topology. Here, we observed a nonlinear relationship between the storage modulus values and the *M*_w_ of the given oTHA. Polymers with the lowest DoO (*e.g*., oTHA3 and oTHA6) retained the largest storage modulus across all oTHAs, albeit their *M*_w_ was only moderately higher than those of oTHA1 or oTHA4 (Table S2). The effective number of cross-links (same DoS of tyramination) and all other conditions remained the same, which suggests that *M*_w_ is the key factor leading to the lower storage modulus in oTHA hydrogels.

**Figure 2:**
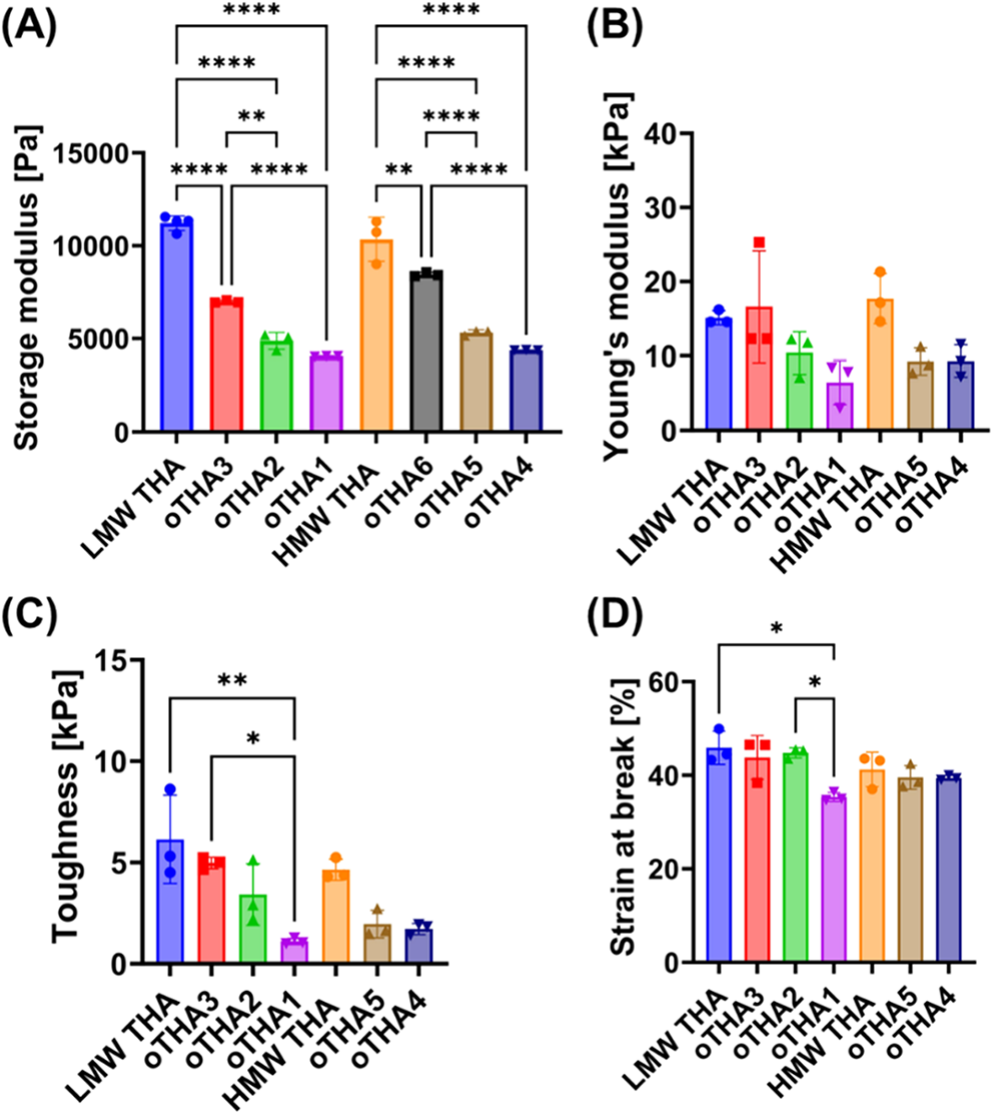
Characterization of 2 w/v% hydrogels (0.1 mM Ru, 5 mM SPS, 23 °C). (A) Storage modulus, shown as the maximum across individual measurements (Figure S13). Data are presented as mean ± SD with individual points (*n* = 3–5 for storage modulus). (B) Young’s modulus, (C) toughness, and (D) strain at break, all measured and calculated in compression testing (*n* = 3). Statistical analysis was done using one-way analysis of variance (ANOVA) with Šídák’s multiple comparisons; significance at *p* < 0.05 (* < 0.05, ** < 0.01, *** < 0.005, **** < 0.001).

To quantitatively assess whether the DoO influenced the mechanical behavior of the hydrogels, compression tests were performed on all samples (excluding oTHA6). From the resulting stress–strain curves, we extracted the compressive Young’s modulus, toughness, and strain at break. A similar trend to that observed in the rheological data was found, with the Young’s modulus decreasing as a function of the DoO (Figure 2B), although this change was not statistically significant. In contrast, a statistically significant decrease in toughness was observed with increasing DoO (Figure 2C), indicating greater brittleness and reduced energy absorption up to the breaking point. This was further supported by the lower strain at break measured for oTHA1 (Figure 2D), corroborating the increased brittleness at higher DoO.

The overall stability of the hydrogels was also significantly affected by the DoO, with all oTHA samples effectively diminishing within 48 h compared to the LMW and HMW THA controls (Figure 3A). The highest DoO oTHA hydrogels all degraded rapidly within 24 h, demonstrating their instability in PBS without hyaluronidase. Subsequently, the indirect cytotoxicity of all formulations was tested according to the ISO-10993-5 guidelines.^67^ For this, all hydrogels at 2 w/v% (0.1 mM Ru, 5 mM SPS) were kept for 24 h in a cell culture medium. The medium containing degraded fragments of all hydrogels was subsequently used as a conditioned medium to test the response of L929 mouse fibroblasts. LDH released into the medium after 24 h of treatment demonstrated no visible cytotoxicity from any of the samples as compared to the control medium, except for oTHA1, which induced a ∼40% cytotoxic response (Figure 3B). Likewise, oTHA4 also demonstrated a nonstatistical trend toward some cytotoxicity with values above 20% (Figure 3B). This effect can likely be attributed to the higher degree of oxidation (DoO) of oTHA1/oTHA4 and the presence of more short-chain degraded fragments (Figure 3A), which may expose unreacted aldehyde groups. These reactive aldehydes can form Schiff-base adducts with amino groups on cell surface proteins or extracellular matrix components, potentially disrupting membrane integrity and leading to the observed cytotoxicity. This was also reflected in the lowest metabolic activity observed for oTHA1 (Figure 3C), despite the similar DNA content measured for it (Figure 3D). Regardless of the lower metabolic activity and DNA content values for all other formulations as compared to the control (Figure 3C,D), the similar ratio of metabolic activity to DNA content indicated that all formulations (except oTHA1) had similarly active cells (Figure 3E).

**Figure 3:**
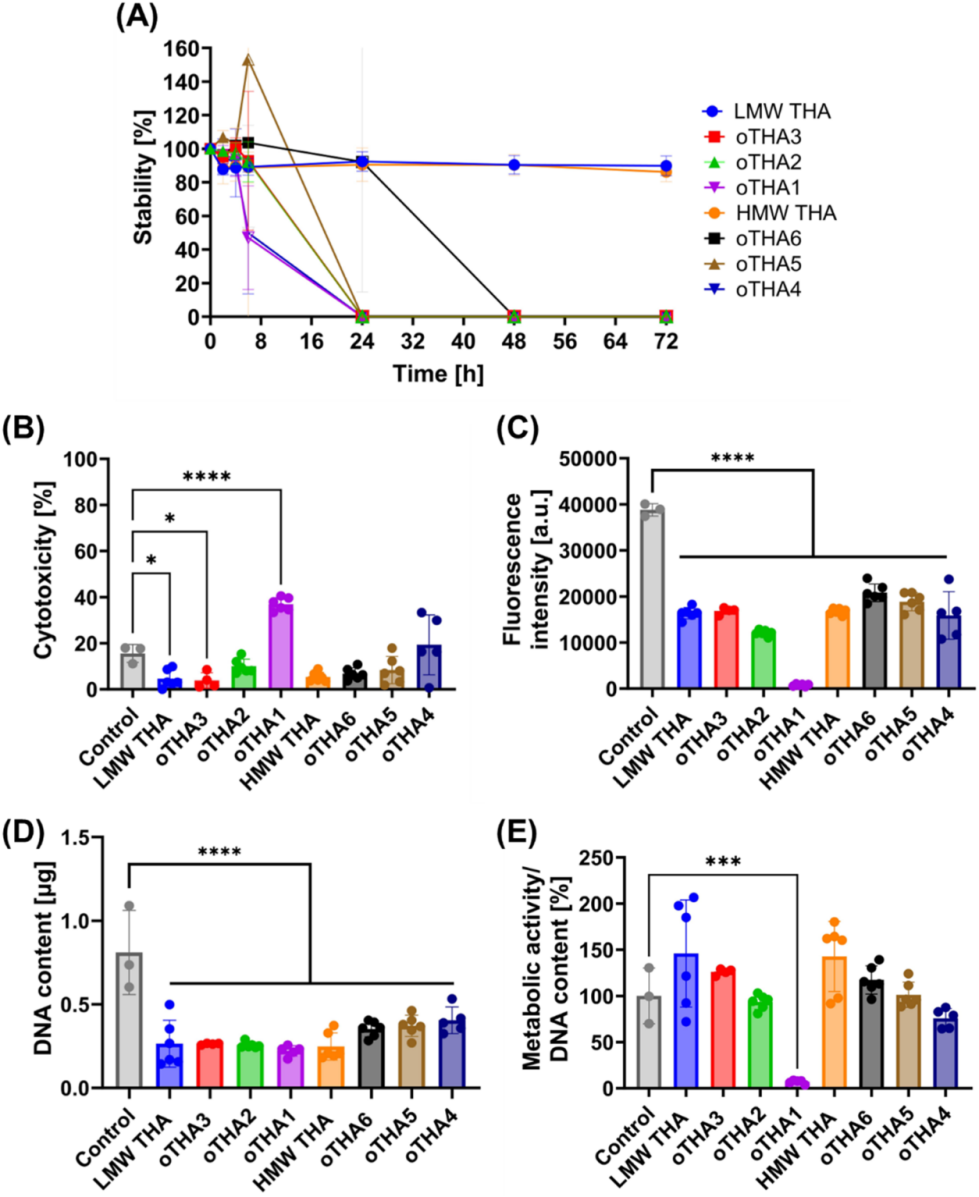
(A) Swelling (stability) rate over 72 h in PBS. Data are presented as mean ± SD with *n* = 6 or *n* = 12. (B–E) Characterization of indirect biocompatibility of 2% w/v hydrogels (0.1 mM Ru, 5 mM SPS, 23 °C). (B) Indirect cytotoxicity of L929 fibroblast cells was measured by LDH release. (C) Metabolic activity of L929 fibroblast cells was measured by CTB. (D) Quantification of DNA content. (E) Ratio of metabolic activity to DNA content, normalized to the average of the control group. (B–D) were measured for cells exposed to the medium from conditioned hydrogels, kept for 24 h during the swelling/stability phase, and after 24 h of exposure to L929 cells (*n* = 3–6 as noted by individually plotted points). Statistical analysis was done using one-way analysis of variance (ANOVA) with Šídák’s multiple comparisons; significance at *p* < 0.05 (* < 0.05, ** < 0.01, *** < 0.005, **** < 0.001).

Overall, the results obtained for oTHA1 and oTHA4 clearly point to the inability to use these samples effectively for any longer-term applications. Hence, in the subsequent sections, we introduce two alternative approaches of hybrid hydrogels: *two-component blended hydrogels*, consisting of both oTHA and THA (Figure 1B), as well as *hydrogel microparticle composites*, where oTHA microgels were embedded in THA networks (Figure 1C). In both cases, we describe the 1:3, 1:1, and 3:1 volumetric ratios of both components and their effects on the viscoelastic, degradation, and adhesive properties. For these hybrid systems, we utilized oTHA2 and oTHA3 as the secondary components mixed with LMW THA, all of which showed no cytotoxicity. The formed hybrid systems are expected to exhibit improved cytocompatibility, as the reactive aldehyde content is diluted within the interstitial THA matrix, yet the two chosen DoO fall within those quoted to be sufficient for adhesion to multiple host tissues.^1^

### 2.3 Combining oTHA and THA in a Two-Component Blended Hydrogel Formulation

Across all our *two-component blended hydrogels*, we noted the retention of a very similar storage modulus (Figure 4A,B), with minimal differences across all groups. For oTHA2, a more pronounced decrease of the storage modulus was observed compared to that of the control LMW THA (Figures 4A and 2A), as expected from the higher DoO for this single-component formulation. Nonetheless, we did not observe any significant differences across the *two-component blended hydrogels* consisting of both components with *G*′_oTHA2:THA 1:3_ = 6300 ± 260 Pa, *G*′_oTHA2:THA 1:1_ = 6300 ± 200 Pa, and *G*′_oTHA2:THA 3:1_ = 5260 ± 420 Pa for oTHA2, and *G*′_oTHA3:THA 1:3_ = 6480 ± 890 Pa, *G*′_oTHA3:THA 1:1_ = 5600 ± 60 Pa, and *G*′_oTHA3:THA 3:1_ = 6080 ± 390 Pa for oTHA3, all falling within similar range of values.

**Figure 4:**
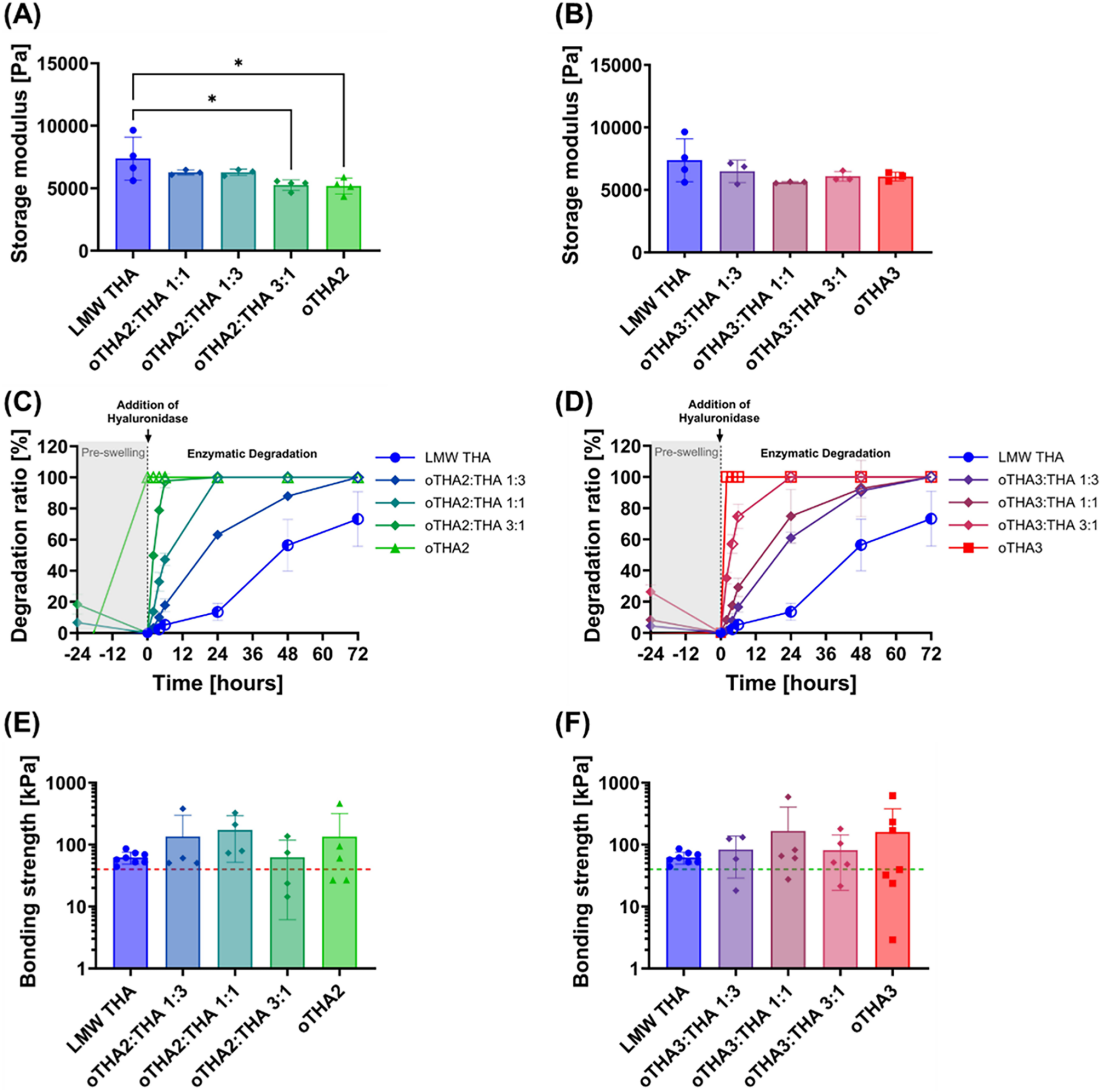
Characterization of two-component oTHA:THA blended hydrogels. (A, B) Storage modulus values (2 w/v% total polymer, 0.1 mM Ru, 5 mM SPS, 23 °C), extracted as maximum across individual measurements (Figure S16), for formulations with (A) oTHA2 or (B) oTHA3 as the secondary component. The same LMW THA control is depicted in both (A, B) graphs. (C, D) Degradation rates of corresponding hydrogels. The addition of hyaluronidase (100 U mL^–1^) is indicated by an arrow. Half-open symbols indicate that the hydrogel broke into two fragments, whereas open symbols indicate the total disappearance/degradation of the hydrogel. (E, F) Bonding strength of hydrogels (3.5 w/v% total polymer, 0.1 mM Ru, 5 mM SPS) to articular cartilage, plotted on a log10 scale to highlight group differences. Horizontal dashed lines depict the benchmark value previously measured for a fibrin glue.^68^ Data are presented as mean ± SD (*n* = 3–7, individual points are depicted). Statistical analysis was done using one-way analysis of variance (ANOVA) with Šídák’s multiple comparisons; significance at *p* < 0.05 (* < 0.05, ** < 0.01, *** < 0.005, **** < 0.001).

Next, the swelling profiles of all formulations, without the presence of any hyaluronidase, were assessed over a 72 h period (Figure S17). The swelling ratio (calculated from samples kept only in PBS, without hyaluronidase) remained stable, fluctuating around 80%, for all formulations except oTHA3:THA 3:1, which showed a peak of 106% at 24 h, followed by a slight decrease to 78% at 72 h (Figure S17C). For oTHA2 and oTHA3 alone, complete degradation in PBS without hyaluronidase occurred within the first 24 h, with oTHA2 degrading faster due to its higher DoO. The minimal shrinkage and lack of extended swelling observed in the *two-component blended hydrogels* are characteristic of HA-based formulations.^69^ This property may mitigate the issues associated with overswelling when such hydrogels are applied as treatments to repair local tissue disorders.^70^

In *two-component blended hydrogels*, increasing the oTHA content proportionally increased the degradation rate in the presence of hyaluronidase (Figure 4C,D). Both oTHA2 and oTHA3 were degraded entirely after the 24 h preswelling step, without requiring hyaluronidase (as shown in Figures 3A and S17). In contrast, the control LMW THA exhibited a steady degradation profile in the presence of hyaluronidase, reaching 73 ± 18% after 72 h, and was the most resistant to degradation (Figure 4C,D). From 0 to 6 h, all other hydrogel formulations showed a linear increase in degradation by hyaluronidase, which then began to level off. This degradation was a function of the formulation, with the oTHA2:THA 1:3 and oTHA3:THA 1:3 formulations being the next most resistant to enzymatic degradation out of all mixtures, remaining intact until complete degradation was observed at 72 h. Naturally, the timing for complete enzymatic degradation has shifted to earlier time points for a higher proportion of oTHA present in the formulations. Furthermore, a clear hallmark was a difference across formulations at a 1:1 ratio, with complete degradation observed for **oTHA2**:THA 1:1 at 24 h and **oTHA3**:THA 1:1 degraded at 72 h due to the difference in DoO (Figure 4C,D). Although the rate of degradation may still seem very high, we note that our tests are a simulation at a high concentration of hyaluronidase (100 U mL^–1^) and do not resemble the real *in vivo* conditions, where values can fall within a 0.015–30 U mL^–1^ range varying across specific locations with different tissues.^71–73^ We, however, expect the trends across these formulations would be maintained in future *in vivo* tests, which are out of the scope of the current work.

Further observational analysis of the degradation process showed that both *two-component blended hydrogels* and their single-component controls (LMW THA or oTHA) underwent a similar *collapse-type mode* of degradation (Figure 4C,D). In this process, the hydrogels gradually softened and spread into a pulp-like state, accompanied by the diffusion of small polymer fragments from the network, without noticeable fragmentation into discrete pieces (Scheme 1(i)). This behavior was characterized by a progressive loss of rigidity and 3D structure, while the overall shape and size gradually decreased over the sampling time during the degradation tests.

**Scheme 1:**
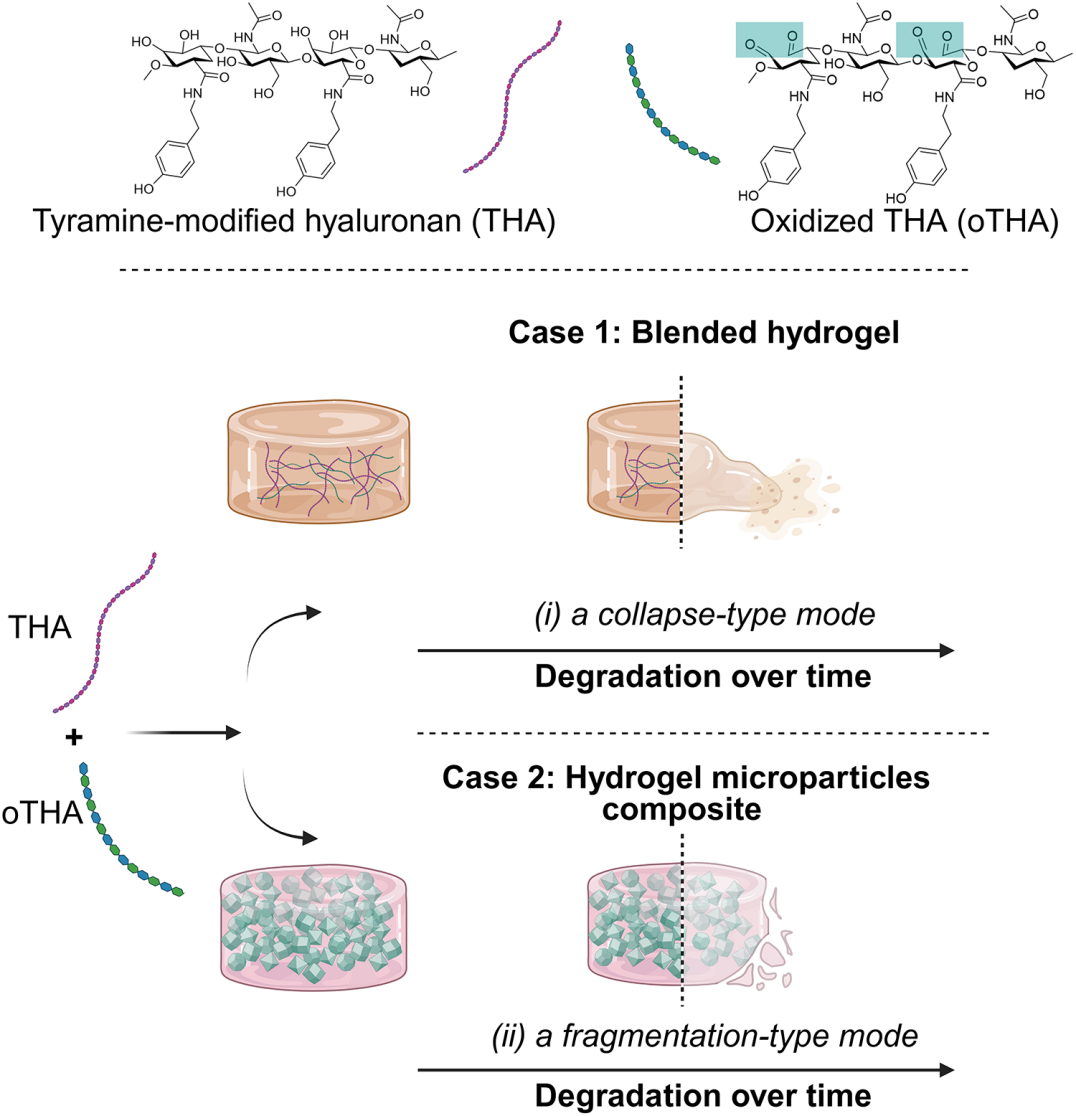
Scheme Depicting Two Distinct Types of Degradation Related to the Underlying Type of Hydrogel. Case 1 Depicts (i) the Collapse-Type Degradation of the Blended Hydrogel, whereas Case 2 Depicts (ii) a Fragmentation-Type Model of Hydrogel Microparticle Composites^a^

a The scheme was created with BioRender.com.

Previously, Raia et al. blended silk fibroin with HA to create hydrogels that balance the strengths of silk, providing mechanical integrity and slow degradation, with HA, which offers enhanced water retention but degrades rapidly on its own.^74^ Pure HA hydrogels were fully degraded by day 6, whereas silk-only gels retained ∼70% of their weight at day 8.^74^ The composites showed intermediate and tunable degradation rates, with higher HA content accelerating mass loss (*e.g*., 10 and 20% HA formulations degraded to ∼50 and ∼15% of their initial weight, respectively, at day 8). This degradation rate, however, was accompanied by different total polymer contents, which influenced the overall viscoelastic properties of the developed networks. In contrast, our approach offers a new, unique method for hydrogel formation at a fixed total polymer content, with closely matched viscoelastic properties, but tunable degradation profiles, effectively using a single parental HA polymer as the starting synthesis material.

Next, the adhesive properties of our formulations were measured by adapting the previously published push-out-test method using bovine cartilage rings (Figure S18).^68^ The rings remained viable within the 48 h culture period, providing a representative ex vivo testing environment for adhesion (Figure S18C). After preliminary attempts to measure the adhesive strength of 2 w/v% hydrogels, we realized that only stiffer formulations, as in the case of previous work,^68^ could be measured. Hence, to match the previously tested THA formulation, we used a higher total polymer content of 3.5% w/v (with hydrogels cross-linked using 0.1 mM Ru, 5 mM SPS). The bonding strength of the control LMW THA hydrogels was 58.8 ± 11.2 kPa (Figure 4E,F), which was higher than the previously benchmarked value for fibrin glue of ∼40 kPa.^68^ Evidently, the use of different cross-linking methods (Ru/SPS versus horseradish peroxidase in the presence of H_2_O_2_) impacted the overall bonding strength of the control, which was effectively 3.8-fold higher than the previously reported value of 15.3 ± 5.8 kPa for 3.5 w/v% THA.^68^ The LMW THA measurements were comparable across 6 repeats with much smaller deviation than that seen for *two-component blended hydrogels* or oTHA controls.

For the oxidized controls (oTHA2 and oTHA3), we observed high variability in the adhesion measurements, with some values exceeding those of LMW THA and others being much lower. This inconsistency can be attributed to several factors. When the hydrogels are cross-linked effectively within the cartilage ring, strong interfacial interactions, putatively assigned to the aldehyde–tissue bonding, enabled intact gel pieces to resist displacement during the push-out test, yielding higher values. In contrast, the partial misalignment of oTHA gels often led to rapid fracture due to their higher inherent brittleness (as depicted in Figure 2C), resulting in lower bonding strength values. Further variability arose from intrinsic donor-dependent differences in cartilage ring geometry, such as thickness and shape variations linked to animal age, as well as from processing steps that, despite a standardized protocol,^68^ may have introduced minor inconsistencies in the ring preparation. Nonetheless, for the *two-component blended hydrogels*, we generally observed a larger proportion of values exceeding the LMW THA average, with indications that formulations containing lower fractions of oTHA were more stable and slightly more adhesive (Figure 4E,F). For example, the average bonding strength increased in the order σ_oTHA2:THA1:3_ = 62.3 kPa < σ_oTHA2:THA1:1_ = 135.5 kPa < σ_oTHA2:THA3:1_ = 172.3 kPa, all above the previously benchmarked value for fibrin glue of ∼40 kPa.^68^ However, the exceedingly large standard deviations and discrepancies across replicates precluded statistical significance. Overall, the combined influence of tissue variability, processing inconsistencies, and brittleness of the control oTHA formulations introduces substantial variation into the adhesion testing, such that these results should be interpreted only as qualitative indications of underlying molecular structure–adhesion relationships, warranting more detailed future investigations.

### 2.4 Combining oTHA Microgels within a THA Matrix–Hydrogel Microparticle Composite Approach

We investigated *two-component blended hydrogels* composed of a fast-degrading component (oTHA) and a slow-degrading component (THA). Varying the proportion of the fast-degrading oTHA fraction influenced degradation and somewhat adhesion, while still allowing the *two-component blended hydrogels* to retain comparable storage moduli. The type of network architecture formed between these two components could be a key determinant of the final set of properties. To test this, we replaced the fast-degrading oTHA component with a pre-cross-linked, well-defined microgels and evaluated the resulting properties of these microgels-embedded networks, named here as *hydrogel microparticle composites*.

First, standard oTHA2 or oTHA3 hydrogels (2 or 3.5 w/v% total polymer, 0.1 mM Ru, 5 mM SPS) were prepared, from which microgels were subsequently generated using an established mechanical fragmentation method with 100 μm grating (see the Section 4).^75^ These microgels were subsequently named oTHA2m or oTHA3m. Due to the use of the mechanical fragmentation method, the obtained oTHA2m and oTHA3m microgels were of irregular shape (Figure S19). From the log-normal distribution fits (Figure S20A,B), we obtained average areas of 12,228 and 15,645 μm^2^ for oTHA2m and oTHA3m, respectively. A perfectly circular microgel of 100 μm in diameter would result in an area of ∼7854 μm^2^, indicating that our microgels were roughly 1.5–2 times larger. The general violin plots demonstrated no significant differences between all obtained microgels (Figure S20C). Both sets of microgels displayed circularity <0.6 (Figure S20D), with oTHA3m showing significantly lower (*p* < 0.0001) circularity. Importantly, we jammed these microgels and evaluated whether they can further cross-link to each other, without any further addition of Ru/SPS (Figure S21). In both cases, the oTHA2m/oTHA3m microgels did not exhibit any further cross-linking without additional photoinitiators, indicating their saturation point after the previous single-component bulk hydrogel preparation.

Next, we evaluated the properties of the *hydrogel microparticle composites* using the same approach as for the *two-component blended hydrogels* (Figure 5). As observed previously, the storage moduli of *hydrogel microparticle composites* remained comparable across all of the ratios used, within the same fixed total polymer concentration of either 2 or 3.5 w/v%, for both oTHA2m and oTHA3m (Figure 5A,5B). In the *hydrogel microparticle composites*, a higher microgel content resulted in greater initial stiffness prior to gelation (Figures S22 and S23). A clear compositional dependence was observed before cross-linking. For oTHAm:THA 1:3, G′ < G″; for oTHAm:THA 1:1, G′ ≈ G″; and for oTHAm:THA 3:1, G′ > G″, both at 2 w/v% (Figure S22) and 3.5 w/v% (Figure S23), in line with the larger fraction of microgels dominating the viscoelasticity prior to cross-linking. The G′ > G″ state observed for *hydrogel microparticle composites* with a larger fraction of microgels (oTHAm:THA 3:1, Figure S23C,G) presents a unique state prior to cross-linking, which is expected to enable the 3D printability of these samples in future studies. For example, the oTHA3m:THA 3:1 microparticle composite was characterized by an *n* = 0.38 ± 0.09 shear-thinning exponent (Figure S24), potentially indicating good downstream printability.^10^ The compressive Young’s modulus, toughness, and strain at break were all largely similar within all tested ratios for both oTHA2m and oTHA3m hydrogel microparticle composite samples (Figure S25), with the only exception of oTHA2m:THA 3:1 showing a statistically significant lower strain at break as compared to all others (Figure S25C).

**Figure 5:**
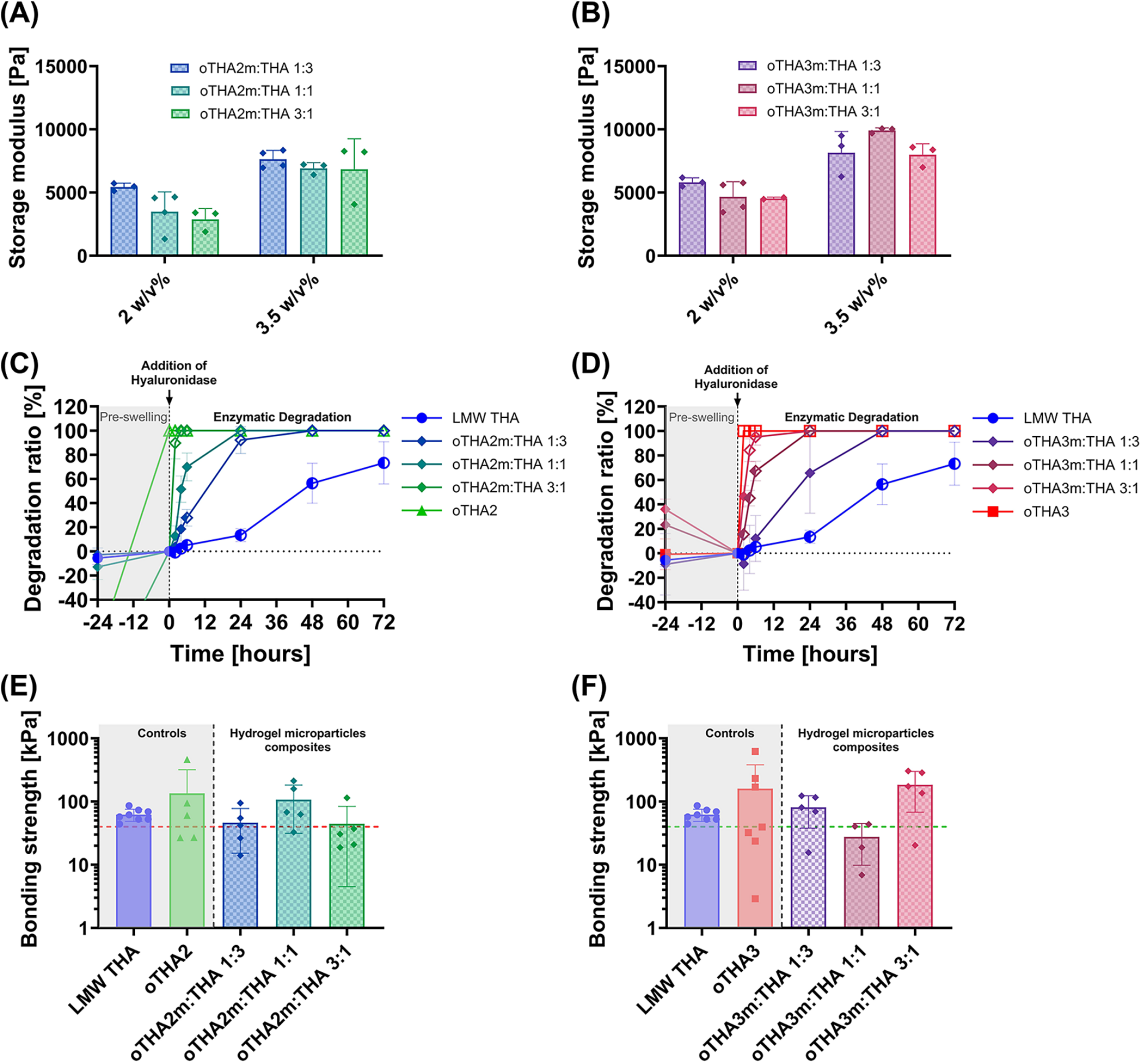
Characterization of oTHAm:THA hydrogel microparticle composites. (A, B) Storage modulus values (2 and 3.5 w/v% total polymer, 0.1 mM Ru, 5 mM SPS, 23 °C), extracted as maximum across individual measurements (Figures S22 and S23), for formulations with (A) oTHA2m or (B) oTHA3m as the secondary component. (C, D) Degradation rates of the corresponding hydrogels (2 w/v% total polymer, 0.1 mM Ru, 5 mM SPS). The addition of hyaluronidase (100 U mL^–1^) is indicated by an arrow. Half-open symbols indicate that the hydrogel broke into two fragments, whereas open symbols indicate the total disappearance/degradation of the hydrogel. (E, F) Bonding strength of hydrogels (3.5 w/v% total polymer, 0.1 mM Ru, 5 mM SPS) to articular cartilage, plotted on a log10 scale to highlight group differences. Horizontal dashed lines depict the benchmark value previously measured for a fibrin glue.^68^ Data are presented as mean ± SD (*n* = 2–8, individual points are depicted). Statistical analysis was done using one-way analysis of variance (ANOVA) with Šídák’s multiple comparisons; significance at *p* < 0.05 (* < 0.05, ** < 0.01, *** < 0.005, **** < 0.001).

The swelling profiles over 72 h of all *hydrogel microparticle composite* formulations, in the absence of hyaluronidase, indicated that the formulations with the largest proportion of microgels (oTHA2/3m:THA 3:1) exhibited greater swelling within the first 24 h, which suddenly decreased, leading to the breakage into fragmented pieces within 48 h (Figure S26). The *hydrogel microparticle composites* with lower fractions of microgels (1:3 and 1:1) retained stable swelling, reaching values of nearly 100 ± 20% at 72 h (Figure S26). In the presence of hyaluronidase, increasing the proportion of oTHAm led to a proportional increase in the degradation rate in both *blended* (Figure 4C,D) and *hydrogel microparticle composites* (Figure 5C,D) with hydrogels completely degraded at 48, 24, and 4 h for higher proportions of oTHA2m, and at 48, 24, and 24 h for oTHA3m formulations, respectively (Figure 5C,D). However, based on the observational analysis during degradation (note the exemplar half-open symbols in the degradation graphs in Figure 5C,D), the *hydrogel microparticle composites* degraded more rapidly than the *two-component blended hydrogels* (Figure 4C,D), displaying a different, *fragmental-like degradation* profile, where individual sections of the hydrogel were “crumbling” off rather than the whole structure progressively collapsing (Scheme 1(ii)). Noting that the relative trends in degradation between the ratios were preserved, we point out that the propagation of degradation could occur largely through the brittleness of embedded microgel particles rather than through the overall diffusive and collapse-like network degradation seen for *two-component blended hydrogels*. We associate this behavior with the larger brittleness (lower toughness) of formulations with higher DoO (Section 2.2 and Figure 2C). The existence of two distinct modes of degradation (Scheme 1) depending on the underlying composite hydrogel architecture could offer an opportunity to choose the desired type of degradation in a plethora of tissue engineering scenarios, where this choice may dictate the release of embedded molecules or enable guided cellular invasion in biological settings.

Given the substantial variability introduced by cartilage tissue heterogeneity and minor inconsistencies in sample processing, we did not observe any clear trends or statistically significant differences in the bonding strength between any of the groups (Figure 5E,F). Notably, *hydrogel microparticle composites* exhibited a lower spread of bonding strength values compared to *two-component blended hydrogels*, which may be attributed in part to their higher initial storage modulus, increasing sample stability prior to the cross-linking step in a cartilage ring. They also demonstrated largely similar or higher values as compared to the benchmark value for fibrin glue of ∼40 kPa.^68^

Overall, while these observations suggest that factors such as the starting rheological properties and hydrogel homogeneity may influence the adhesion measurement outcomes, the multitude of overlapping variables preclude us from drawing clear conclusions regarding the adhesion differences between *hydrogel microparticle composites* and *two-component blended hydrogel* formulations, necessitating more extensive testing in the future.

Finally, we performed confocal microscopy imaging of the *hydrogel microparticle composites*, where the interstitial THA matrix was labeled with 0.1 wt % fluorescein isothiocyanate 2 MDa dextran to limit its diffusivity (Figure 6A–F). The assumption here was that labeled (bright) regions depicted the interstitial THA matrix, whereas empty (dark) areas comprised regions that contained microgels or hydrated space. The measurements corresponded to the proportion of the overall THA matrix, with brighter regions detected for the oTHAm:THA 1:1 and oTHAm:THA 3:1 ratios (Figure 6G,H), clearly showing more matrix space between the interstitial THA matrix (Figure 6B,C,6E,F). Visually, no significant differences were observed between the oTHA2m and oTHA3m groups, indicating similarities in the topological network composition. This important similarity may offer opportunities for the modulation of future similar cellular responses with an intrinsically different programmed degradation rate.

**Figure 6:**
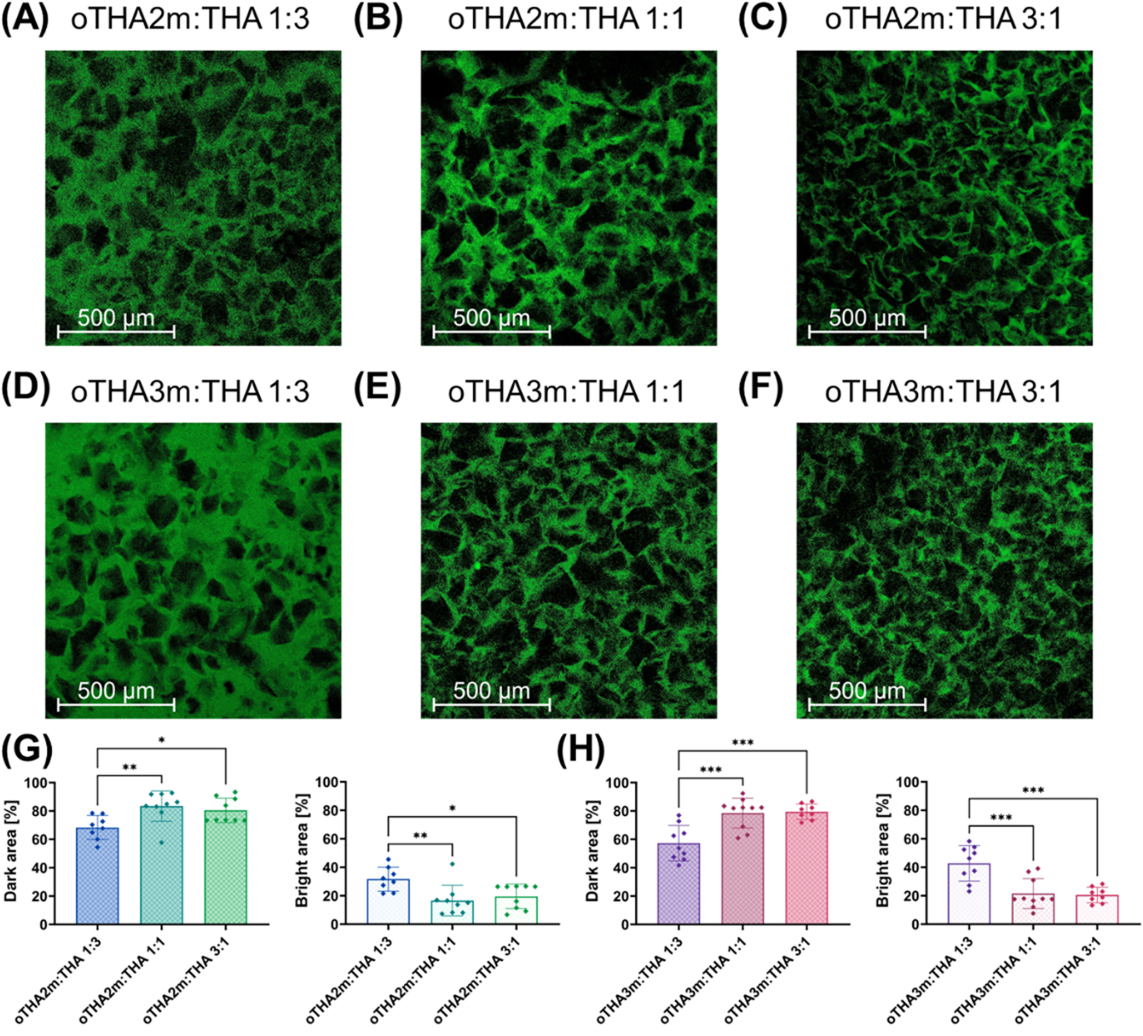
Interstitial matrix volume measurements in oTHAm:THA hydrogel microparticle composites for (A–C) oTHA2m series and (D–F) oTHA3m (2 w/v% total polymer, 0.1 mM Ru, 5 mM SPS). 0.1 wt % fluorescein isothiocyanate–2 MDa dextran was added to the THA part of the matrix after gelation and subsequent imaging. Dark and bright areas were calculated across *n* = 8–10 images for (G) oTHA2m:THA and (H) oTHA3m:THA hydrogel microparticle composites.

## 3 Conclusions

Our study demonstrates that the degree of oxidation in oTHA is a critical determinant of hydrogel performance, directly influencing mechanical stability, degradation, brittleness, and tissue adhesion (Scheme 2). By combining oxidized and nonoxidized THA into a hybrid hydrogel, either in a *two-component blended* or *hydrogel microparticle composite*, we can decouple the storage modulus from degradation and adhesion, enabling more precise tuning of the resulting biomaterial properties (Scheme 2). *Two-component blended hydrogels* exhibit gradual, collapse-type degradation, whereas *hydrogel microparticle composites* with embedded microgels degrade via abrupt fragmentation, highlighting how molecular design and hydrogel architecture can control degradation modes (Scheme 1). These findings underscore the potential of oTHA–THA hydrogels, derived from the same parental HA biopolymer, as versatile platforms for tissue engineering, where programmable mechanical properties, adhesion, and degradation can be leveraged to guide cell behavior and tissue formation, as depicted in Scheme 2. Ultimately, the ability to independently tailor the adhesion, degradation rate, and mechanical properties of networks provides a strategic framework for designing new types of hydrogels with application-specific functionality in regenerative medicine.

**Scheme 2:**
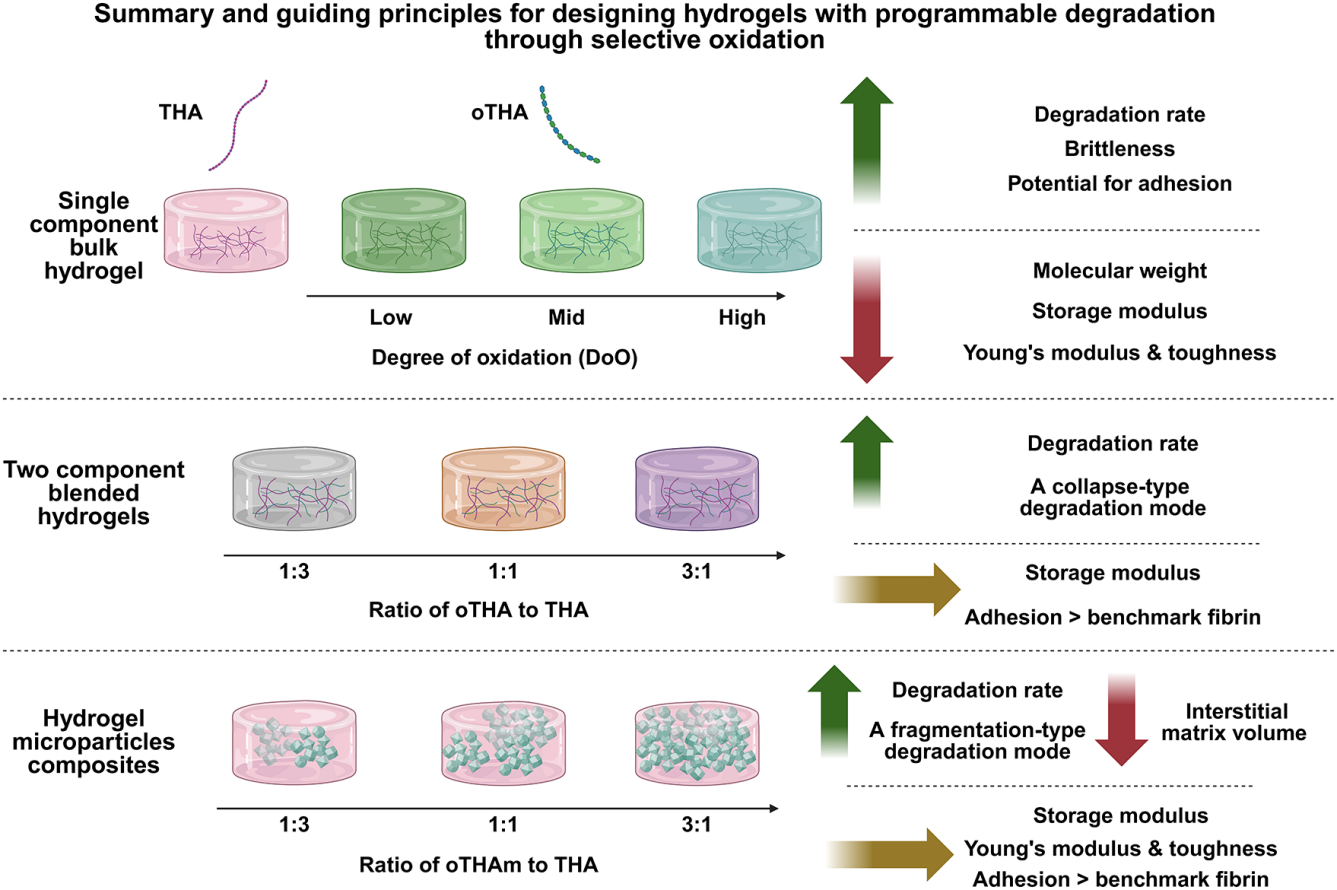
Scheme Summarizing and Guiding Principles for Designing Hydrogels with Programmable Degradation through Selective Oxidation^a^

a The key findings are depicted using schematic arrows for increased/improved/gained ↑; decreased ↓; or largely remaining similar →. The scheme was created with BioRender.com.

## 4 Materials and Methods

### 4.1 Synthesis and Characterization of Tyramine-Modified Hyaluronan (THA)

The tyramination of hyaluronan (HA, Contipro Nutrihyl) of two molecular weights (*M*_w_): ∼280–290 kDa (abbreviated as LMW THA), or ∼1.64 MDa (abbreviated as HMW THA) was carried out with tyramine hydrochloride (Tyr HCl) using a previously established protocol.^46,56,76^ In brief, HA sodium salt (1 w/v%) was dissolved in ultrapure water (Mili-Q, EMD Milipore) before being heated to 37 °C. Tyramine-modified HA (THA) was then prepared in a one-step reaction by the addition of 1.25 mmol of 4-(4,6-dimethoxy-1,3,5-triazin-2-yl)-4-methylmorpholinium chloride (DMTMM, TCI Europe, purity: >95%) coupling agent, and subsequently, 1.25 mmol tyramine hydrochloride (Roth, purity: ≥98%) was added dropwise to the solution. The reaction was carried out at 37 °C under continuous stirring for 24 h. The product was purified via precipitation with 96% ethanol (EtOH, Honeywell Fluka, ≥99.8% stock solution) after the addition of 10 v/v% saturated sodium chloride (NaCl, Roth, purity: >99.5%). Several wash steps were performed until the argentometric test with silver nitrate (AgNO_3_; Honeywell Fluka, purity: ≥99.5%) was negative, indicating that no NaCl remained. The product was vacuum-dried for 72 h, with the first 24 h at 25 °C, and then for two final days at 40 °C. For HMW THA, purification and washing were repeated twice to ensure the complete removal of trapped H_2_O and ethanol. Ultraviolet (UV) absorbance measurements at 275 nm on an Infinite 200 Pro plate reader (Tecan, Männedorf, Switzerland) were performed to confirm the degree of substitution (DoS) of tyramine on THA. The average DoS obtained across all THA batches was 5.72 ± 0.26%. The loss of drying (LOD) was also evaluated. For this, 100 mg of THA was weighed into a glass vial and dried under vacuum at 105 °C for 24 h. LOD was then calculated using the formula

1 $$\mathrm{LOD}=\frac{W_{b}-W_{a}}{W_{b}}\times 100\%$$

where *W*_b_ is the weight of THA before drying, and *W*_a_ is the weight of THA after drying. The average LOD obtained across all THA batches was 7.94 ± 3.82%.

### 4.2 Synthesis of Oxidized Tyramine-Modified Hyaluronan (oTHA)

The synthesis of oxidized tyramine-modified hyaluronan (oTHA) was adapted from previous work.^1,61^ Briefly, as-synthesized THA was oxidized by using sodium periodate (NaIO_4_, Sigma-Aldrich, purity: ≥99.8%). For synthesis, 1 g of THA (either LMW or HMW) was dissolved in 45 mL of ultrapure Milli-Q water at 4 °C under stirring at 12 rpm overnight. The following day, the solution was transferred to conical flasks and adjusted to a concentration of 10 mg mL^–1^. NaIO_4_ was freshly prepared at a concentration of 90 mg mL^–1^ and then added dropwise to the THA solution in specific ratios of 1:1, 2:3, and 1:3 (NaIO_4_:THA), depending on the desired degree of oxidation (DoO); see Tables 1 and 2. As explained in the main text, 3 sets of reactions were performed (Set A, Set B, and Set C). Set A and Set B followed the details in Table 2, whereas Set C was 3-fold increased in all amounts to produce higher amounts of biopolymers and was performed exclusively on LMW THA (DoS: 5.6 ± 0.1%). By adjustment of the molar ratio of sodium periodate to THA, three different DoOs were obtained. The reaction proceeded under stirring (100–200 rpm) for 2 h at room temperature in the dark, after which it was quenched by the addition of ethylene glycol (Sigma-Aldrich, purity ≥99%). To remove unreacted compounds and small molecular byproducts, the obtained product was dialyzed against ultrapure water for 7 days using an approximate volume ratio of 1:50 (oTHA to H_2_O), with 5 water changes across this period. For the first synthesis, we used 12–14 kDa dialysis membranes (Spectra/Por Dialysis Tubing), which were later replaced with 3.5 kDa (Spectra/Por Dialysis Tubing) membranes for subsequent batches of LMW oTHA. Finally, the dialyzed samples were freeze-dried for 7 days. In Section 2.2, we focus on the comparison of single-component hydrogels generated from reaction sets A and B. The syntheses from set C were used for the results presented in Sections 2.3 and 2.4. Due to the reproducibility across syntheses for all individual reaction sets A–C and the use of scaled-up reactions, in Sections 2.3 and 2.4, we continue describing samples using only the oTHA2/3 description without the annotation to set C anymore. The molecular structures of THA and oTHA presented in Figure 1A were drawn by using ChemDraw 18.2. The ACS document 1996 style sheet was used for drawing settings.

### 4.3 Measurement of the Degree of Oxidation (DoO) by the Purpald Colorimetric Assay

The DoO was measured by adapting the Purpald assay from ref 77. For this, oTHA samples with various levels of oxidation (and controls of THA and ethylene glycol) were dissolved in 1 M NaOH (Sigma-Aldrich, purity: ≥97%) at a concentration of 0.5 mg mL^–1^, and loaded in triplicate into a 96-well plate (Greiner Bio-One, UV-Star microplate, 96-well, COC, F-bottom/chimney well). A standard curve was generated from a serial dilution of propionaldehyde (Sigma-Aldrich, purity: ≥97%) in NaOH, ranging from 15 to 70 μg mL^–1^. The Purpald reagent (Sigma-Aldrich, purity ≥99%) was freshly prepared at 10 mg mL^–1^ and added in a 1:1 ratio to each well. The plates were then incubated in ambient air for 24 h. NaOH with and without Purpald reagent served as additional controls. Absorbance was measured at 550 nm on an Infinite 200 Pro plate reader (Tecan, Männedorf, Switzerland) with three technical repeats. In each disaccharide unit of THA, there were 2 available free −OH groups. Based on the standard curve, the number of aldehyde groups (free −OH groups) in each sample was quantified. The THA measurements were subtracted from the oTHA to yield the reported DoO. For each synthesized batch, we performed two replicates of all plate measurements (*n* = 2) with *n* = 3 technical replicates each within 3 days of obtaining the freeze-dried oTHA. These measurements were also performed over time (up to 30 days) to confirm the stability of the product stored in ambient conditions over time, which yielded no measurable difference in the obtained DoO.

### 4.4 Proton Nuclear Magnetic Resonance (^1^H NMR) Spectroscopy

For NMR analysis, THA or oTHA samples were prepared at 3 w/v% and dissolved in 1 mL of 0.04 w/w% hyaluronidase in D_2_O at 37 °C and left overnight for full dissolution, before measurement on ^1^H NMR (300 MHz, Bruker Avance III), taken at room temperature with 128 scans.

### 4.5 Attenuated Total Reflectance–Fourier-Transform Infrared (ATR-FTIR) Spectroscopy

Freeze-dried, as-synthesized THA/oTHA powders were placed onto the crystal surface of a Jasco FT/IR-4X spectrometer equipped with a diamond multibounce attenuated total reflectance (ATR) plate. The transmittance spectra were recorded (128 scans) between 4000 and 400 cm^–1^ with a resolution of 2 cm^–1^. Air was used as a background before each measurement and was automatically subtracted from the recorded spectra using the software provided with the instrument. From all scans, the region of 900–1800 cm^–1^ was analyzed by normalization to the highest peak (at ∼1032 cm^–1^) to show the differences within the aldehyde peak at 1732 cm^–1^. The following ratios: aldehyde *vs* C–O–C stretching (*I*_1732_/*I*_1610_), aldehyde *vs* amide II (*I*_1732_/*I*_1557_), aldehyde *vs* C–H bending (*I*_1732_/*I*_1370_), and aldehyde *vs* C–O stretching (*I*_1732_/*I*_1030_), were all calculated by taking the maximum intensity value (within ± 10 cm^–1^) of the key denoted peaks after normalization of the spectrum and are presented in Table S1 for all reactions.

### 4.6 Size-Exclusion Chromatography—Multiangle Light Scattering (SEC-MALS) Measurements of Molecular Weight

All SEC-MALS characterizations were performed in the Laboratory of Molecular and Rheological Characterization of the Istituto di Scienze e Tecnologie Chimiche “G. Natta” (SCITEC) of Consiglio Nazionale delle Ricerche (CNR) Via A. Corti 12 I-20133 Milano (Italy). SEC-MALS characterization was performed on one set of THA samples: (i) LMW THA synthesized from HA with an average *M*_w_ = 280 kDa and (ii) HMW THA synthesized from HA with an average *M*_w_ = 1.64 MDa. From each THA, three samples were obtained by oxidation (oTHA) using different ratios of NaIO_4_, as described above. Sample solutions were obtained by dilution in the SEC mobile phase at the established concentration and allowed to rest for 24 h to obtain maximum solubility. All solutions before injection into the SEC-MALS chromatography system were filtered by using 0.45 μm pore size Nylon filters. The molecular weight distribution (MWD) of the samples was obtained using a modular SEC system. The SEC system consisted of an Alliance 2695 separation module from Waters equipped with two online detectors: (i) MALS Dawn DSP-F photometer from Wyatt; and (ii) 2414 differential refractometer (DRI) from Waters as a concentration detector. The setup of the multidetector SEC system was serial in the following order: Alliance → MALS → DRI. In a multidetector SEC system, different online detectors have an intrinsic temporal delay because they are located at different positions in the fluidic path. Consequently, the alignment of different signals was needed. The experimental methodology for the consistent use of the SEC-MALS system has been described before.^78,79^ The following experimental conditions were used:

- Mobile phase: 0.2 M NaCl
- Column set: 2 TSKgel PWXL (G6000–G5000: 13 and 10 μm particle size, respectively) from Tosoh Bioscience (D)
- Flow rate: 0.5 mL min^–1^
- Temperature: 35 °C
- Injection volume: 150 μL
- Sample concentration: ∼0.5 mg mL^–1^ (for HMW samples) and 1.0 mg mL^–1^ (for LMW samples)
- Degassing: Vacuum

The MALS used is an elastic or total intensity light scattering detector, which uses a vertically polarized solid-state laser (λ = 664 nm), and simultaneously measures the intensity of the scattered light at 18 fixed angular locations ranging from 13.04 to 157.72°in the aqueous solvent. A MALS detector coupled to a concentration detector allows determining the absolute molecular weight (*M*_w_) and the size, *i.e*., the root-mean-square radius ⟨s2⟩^1/2^, denoted as the radius of gyration (*R*_g_), of each eluting fraction. The MALS calibration constant was calculated using ultrapure toluene solvent, assuming a Rayleigh factor *R*(θ) = 1.406·10^–5^ cm^–1^. Angular normalization of different photodiodes was performed by measuring the scattering intensity in the 0.2 M NaCl mobile phase of a bovine serum albumin globular protein (BSA: *M*_w_ = 66.4 kDa, *R*_g_ = 2.9 nm), assumed to act as an isotropic scatterer. We followed the use of an MALS detector, as described in the literature.^78,79^ We assumed that the specific refractive index increment with respect to the 0.2 M NaCl solvent (d*n*/d*c*) of all samples was the same as that of native HA (0.15 mL g^–1^).^80–82^ The HPLC/SEC chromatographic software used for chromatogram acquisition and SEC data elaboration was Empower Pro 1.0 from Waters. The MALS software for light scattering data acquisition and data analysis was Astra 7.3.2.19, from Wyatt.

### 4.7 Molecular Mass Measurements Utilizing Dynamic Light Scattering (DLS)

To evaluate *M*_w_ and hydrodynamic diameter (*d*_hyd_), a series of serial dilutions of THA and oTHA samples was prepared in 0.02 μm-filtered (Sigma-Aldrich, inorganic membrane filter, Whatman WHA68092002) phosphate-buffered saline (PBS) and analyzed using a Litesizer DLS 500 (Anton Paar GmbH, Austria) connected to a computer with Kalliope software (v3.8.2, Anton Paar GmbH, Austria). Before sample preparation, the purity of the PBS solution was verified by measuring *d*_hyd_ (*n* = 3). The detection of particles of any size was an indication of contamination, and the PBS solvent was discarded and replaced with freshly filtered PBS. For molecular mass measurements of samples from the original LMW HA (∼280–290 kDa, Contipro Nutrihyl, 041218-E1), an 8 mg mL^–1^ stock solution was prepared in filtered PBS, dissolved overnight, and diluted to the following concentrations: 4, 2, 1, 0.5, 0.25, 0.1, and 0.05 mg mL^–1^. For samples synthesized from HMW HA (∼1.64 MDa, Contipro Nutrihyl, no. 30101113), a 4 mg mL^–1^ stock solution was prepared and diluted to the following concentrations: 2, 1, 0.5, 0.25, 0.1, and 0.05 mg mL^–1^. For each dilution, filtered PBS was pipetted into 1.5 mL Eppendorf tubes, followed by the appropriate volume of the stock solution, and gently vortexed. Immediately after dilution, 1 mL of the highest concentration sample was transferred into a freshly cleaned quartz cuvette (Anton Paar, 12.5 mm × 12.5 mm × 45 mm, 163390), sealed with a plastic lid, and inserted into the instrument. Molecular mass measurements were performed using the following parameters: side scatter angle (90°), temperature of 25 °C, equilibration time of 1 min, and d*n*/d*c* = 0.15 mL g^–1^, using PBS as solvent with refractive index (RI) = 1.3318 and toluene as reference (RI = 1.4925). A coil-shape correction factor^83^ was applied with *d*_hyd_ values chosen based on prior DLS measurements across the full concentration series (Figures S6–S12) or from the literature.^84^ Each dilution was measured twice, with the cuvette rotated 90° around its vertical axis between the measurements. After the second measurement, the next lowest concentration was analyzed. The overall measurement series started with the highest concentration and continued in descending order, ending with the solvent and reference control measurements. Prior to sample analysis, the 0.02 μm-filtered (Sigma-Aldrich, inorganic membrane filter, Whatman WHA68092002) anhydrous toluene (Sigma-Aldrich, purity: >99.8%) reference was inspected for clarity and cleanliness using the same methodology as for the quality control of PBS. After each measurement, the quartz cuvette was thoroughly rinsed with Milli-Q water, followed by isopropanol (59300, Sigma-Aldrich) and gently dried with compressed air. Before the first and following final measurements, the cuvettes were cleaned with 2 v/v% Hellmanex III (Hellma Analytics) detergent prepared in Milli-Q H_2_O. Hydrodynamic size (DLS) measurements were performed for each dilution step individually. For both molecular mass and hydrodynamic size measurements, the samples were prepared freshly and discarded after use. Static light scattering (SLS) provided Debye plots, from which approximate average *M*_w_ values were obtained. First, a series of DLS measurements across all concentrations used was performed to establish the average hydrodynamic size *d*_hyd_ and investigate the polydispersity of the samples (Figures S6–S12). Further details are provided in Section S2 of the Supporting Information.

### 4.8 Preparation of Single-Component Hydrogels Using Ruthenium (Ru) and Sodium Persulfate (SPS) Photocross-linking

Hydrogels were either prepared at 2 w/v% or at 3.5 w/v% polymer content, and at 0.1 mM tris­(bipyridine)­ruthenium­(II) chloride (Ru, Combi-Blocks, purity >99%) and 5 mM sodium persulfate (SPS, Sigma-Aldrich, purity: ≥98%), and used for experiments as described in the main text. First, THA or oTHA powder was dissolved in 0.22 μm-filtered PBS, gently vortexed, and left on a rotating wheel in a dark, cold room (4 °C) at 5 rpm overnight. The following morning, a freshly prepared stock solution of Ru solution was added to the dissolved polymer, gently vortexed, and returned to the cold room for approximately 30 min. Finally, the freshly prepared SPS was added to the final hydrogel precursor solution, gently vortexed, and placed back on the rotating wheel in the cold room for an additional 15 min. All formulations were protected from light during the procedure. To ensure full homogeneity, the hydrogel precursor was further mixed using a pipette tip prior to insertion into the PDMS molds. Molds with either a diameter of 15 mm and a height of 5 mm or a diameter of 6 mm and a height of 3 mm were used, depending on the desired experimental setup. Photocross-linking was then initiated by exposing from both the top and bottom of the mold to a 450 nm LED light source (SST-10-B, Luminus) at an intensity of 5 mW cm^–2^ for 3 min on each side. This power was achieved by maintaining the distance between the LEDs and the sample at 36 cm. The fully formed hydrogels were carefully extracted from the molds using a spatula.

### 4.9 Preparation of Two-Component oTHA/THA Blended Hydrogels

For the two-component blended hydrogels, separate stock solutions of oTHA and THA were initially prepared by dissolving each polymer in PBS as described above. The following morning, the two solutions were mixed in volume-to-volume ratios of 1:3, 1:1, and 3:1 (oTHA:THA). Subsequently, 0.1 mM Ru and 5 mM SPS were added to each formulation, as described above for the single-component hydrogels. Photocross-linking was performed in the same way as described above.

### 4.10 Preparation of oTHA Microgels

First, the 2 or 3.5% w/v oTHA (0.1 mM Ru and 5 mM SPS) single-component bulk hydrogel was prepared and photocross-linked in a PDMS mold with wells of 15 mm diameter and 5 mm height, as described above. Typically, 8–10 oTHA photocross-linked hydrogels were then extracted from the molds by first gently loosening the edges with a spatula. The tip of the spatula was slid beneath the hydrogel to lift it from the mold. All hydrogels were then collected in a single 10 mL plastic syringe and extruded through a 100 μm cell strainer into a second 10 mL syringe, as described previously.^75^ This process was repeated twice using a larger Falcon tube (50 mL) as the final collection container instead of a syringe. The same cell strainer was used for all three extrusion steps, except in cases when it was damaged, and was subsequently replaced. Subsequently, PBS was added at a volume ratio of 2:1, followed by centrifugation of the PBS-microgel mixture at 4700 rpm for two consecutive 5 min cycles (with the steepness setting set to 9). As not all microgels could be compressed into a pellet, both the microgel suspension and pellet were additionally vacuum filtered using a DURAN filter funnel (Sigma-Aldrich, porosity: 2). During filtration, the microgel paste was mixed intermittently and flattened to ensure uniform drying. The vacuum pressure was turned off once a thin surface crust began to form, indicating the onset of dehydration. The drying process would typically begin within 2–5 min after filtration of the vacuum, depending on the volume of the sample. Subsequently, the microgels were either immediately used to formulate the hydrogel microparticle composites (section below) or collected and stored in a fridge at 4 °C until further use. The number of hydrogels used (volume/no. of photocross-linked hydrogels) was adjusted as needed for different experiments, and the whole protocol was followed in the same way. The formation of microgels following this protocol is volume-independent.

### 4.11 Preparation of Hydrogel Microparticle (oTHA Microgel-Embedded) Composites

In this step, we prepared a hydrogel consisting of oTHA microgels embedded in an interstitial THA matrix, henceforth referred to as a *hydrogel microparticle composite*. Concurrently with the mechanical fragmentation of the oTHA microgels described above, the THA interstitial matrix precursor was prepared. The final precursor solution consisted of a dissolved 2 w/v% THA polymer in PBS, supplemented with 0.1 mM Ru and 5 mM SPS. Ru was added prior to mechanical fragmentation of the oTHA hydrogels, while SPS was introduced into the Ru-containing THA solution following the initiation of centrifugation of the oTHA microgels. The intermediate solution and final matrix precursor were returned to the cold room for continuous rotation at 5 rpm following each mixing step. All components were protected from light, and the assembly of the precursor was performed behind an aluminum-covered hood. For the fabrication of *hydrogel microparticle composites*, oTHA microgels were weighed in aluminum-covered Eppendorf tubes according to predefined volume ratios of 1:3, 1:1, and 3:1 (oTHA microgel:THA matrix). Subsequently, the corresponding volume of the THA precursor (containing Ru and SPS) was added to each tube. To ensure complete homogenization, the components were gently vortexed for 5 s and then manually mixed using a pipette tip. These formulations were subjected to secondary photocross-linking under 450 nm light for 6 min, as described in the preparation of single-component hydrogels section. Hydrogels prepared in oTHA microgel:THA matrix ratios of 1:3 and 1:1 were generally transferred using a positive displacement pipette, while those with a 3:1 ratio required the use of a small spatula for homogenization and final extraction from the Eppendorf tube.

### 4.12 Rotational Rheology and Oscillatory Photorheology

Rheological measurements were performed by using an Anton Paar MCR-302 rheometer (Anton Paar GmbH, Austria) equipped with a Peltier temperature control system and a light source (X-Cite 200DC fluorescence illuminator, Lumen Dynamics Group, Canada). Rotational experiments were performed to evaluate viscosity changes due to the oxidation reaction, whereas oscillatory measurements were performed to study the kinetics of hydrogel formation and provide their mechanical properties.

To evaluate the viscosity and effects of the oxidation reaction, THA and oTHA were left rotating overnight in a cold room at 4 °C at 2 w/v% in PBS until dissolved. A cone plate geometry of 1° (CP-25 plate, 25 mm diameter) with a gap distance of 0.049 mm was then used for *n* = 3 or *n* = 4 repeats in a flow curve experiment within the range 0.1–1000 s^–1^, with samples being held at each shear rate until a stable reading was reported by the instrument. A 60 s waiting time was applied prior to the measurement. The samples were gently pipetted onto the stage prior to the measurements. The measurements were taken at 23 °C. From the flow curves, viscosity values at 10 s^–1^ were taken across all formulations for a qualitative comparison of the oxidation and overall indirect confirmation of the success of the oxidation reactions.

To study the kinetics of gel formation, oscillatory time sweep measurements were performed at a fixed frequency of *f* = 1 Hz, a constant strain of ε = 0.2%, and a time interval per point of 2.5 s for a total duration of 300 s. All measurements were conducted at 23 °C with a plate–plate (PP-25 plate, 25 mm diameter), a fixed gap size of 1 mm, and an initial equilibration period of 60 s before the start of the measurement. For each measurement, 0.55 mL of the hydrogel precursor solution (either 2 w/v% or 3.5 w/v%, as described in the preparation section) was applied to the lower plate. The measuring system was then gently lowered, and any excess material was removed. Twenty-five seconds after the measurement began (excluding the initial waiting time), the light source was switched on and remained active until the end of the experiment. Illumination was performed using the full spectrum of white light at an intensity of 5 mW cm^–2^. Each formulation was tested in triplicate (*n* = 3). The gelation point, defined by the crossover of the storage (G′) and loss (G″) moduli (*i.e*., G′ > G″), was aligned at the 25 s mark to ensure comparability between replicates and formulations. For subsequent analysis, the mean and standard deviation of G′ and G″ over time were calculated and plotted as point-line graphs. Furthermore, the maximum G′ and the corresponding G″ values at that time point were evaluated across the samples.

To evaluate the shear thinning of the oTHA3m:THA 3:1 sample, a plate–plate geometry (PP-25 plate, 25 mm diameter) with a gap distance of 1 mm was used for *n* = 2 repeats in a flow curve experiment within the range 0.001–1000 s^–1^, with samples being held at each shear rate until a stable reading was reported by the instrument. A 60 s waiting time was applied prior to the measurement. Samples were gently pipetted onto the stage prior to measurement at 23 °C. The shear-thinning behavior was characterized by fitting the power law equation of the shear rate–viscosity rheology plot

2 $$\eta =K{\mathrm{\gamma ̇}}^{n-1}$$

where η is the viscosity, γ̇ is the shear rate, *K* is a consistency value, and *n is* the shear thinning exponent. For all rheological tests, silicone oil (Sigma-Aldrich) was used to seal the edges and prevent the sample from drying out prior to the start of the measurement.

### 4.13 Compression Testing

Hydrogel samples (2 w/v% total polymer content, 0.1 mM Ru, 5 mM SPS) were prepared as described above. Briefly, the solutions were placed in a preprepared PDMS mold with a cylindrical shape of 5 mm height and 15 mm diameter, and photocross-linked as described above. Uniaxial compression tests were performed on an electrodynamic testing system (LTM1, ZwickRoell) equipped with a 50 N load cell (U9C, HBM). The testing protocol consisted of an initial 0.1 N preload, followed by compression until breaking (with a maximum set to 60% strain equivalent to 3 mm) at a rate of 1 mm min^–1^ (0.33% s^–1^). Data acquisition was performed at a sampling rate of 50 Hz. Stress–strain graphs were plotted, and the compressive modulus for each sample was calculated from the linear portion of the obtained curves. The breaking point was then manually identified near the maximum value found within the measurements, and the toughness, representing the area under the curve, was then calculated using a trapezoidal rule. For all tests, 2 or 3 independently prepared samples (*n* = 2 or *n* = 3) were measured.

### 4.14 Swelling and Degradation Assays

For swelling and degradation studies, the hydrogel precursor solutions were cast and photocross-linked as described before in PDMS molds with a diameter of 6 mm and a thickness of 3 mm. The freshly prepared gels were weighed using an electronic Semi-Micro Balance (Mettler Toledo) before being transferred to TPP tissue culture plates (Sigma-Aldrich), immersed in 2 mL of PBS, and incubated at 37 °C to allow swelling. At predefined time points (2, 4, 6, 24, 48, and/or 72 h), the hydrogels were dried with tissue paper, weighed to determine their wet weight (*W_t_)*, and subsequently returned to the incubator. To handle fragile samples, a combination of tweezers and a spatula was used. The tweezers held the hydrogel in place on the spatula to prevent sliding back into the PBS during transfer. Any fragmentation, ranging from partial to complete disintegration, was recorded and represented in the plots as half-filled or open symbols, respectively. Measurements were stopped when the hydrogel could no longer be retrieved (indicated by 0 in the plot). The swelling ratio (also noted as *stability*) at each time point was calculated as follows

3 $$\mathrm{swelling}\;\mathrm{ratio}=\frac{W_{t}}{W_{0}}\times 100\%$$

where *W*_*t*_ is the wet weight at time *t*, and *W*_0_ is the initial weight after photocross-linking.

For the degradation assay, the hydrogels were first allowed to swell in PBS for 24 h at 37 °C. The samples were weighed only at the beginning and end of this equilibrium step, marking the starting weight of the sample (*W*_swollen_). Following the second measurement, the hydrogels were transferred to new multi-well plates and immersed in 2 mL of PBS containing 100 U mL^–1^ hyaluronidase (Sigma-Aldrich). The samples were then incubated at 37 °C for an additional 72 h. At defined time points (2, 4, 6, 24, 48, and 72 h), the hydrogels were removed from the enzyme solution, briefly blotted to remove excess liquid, and weighed to assess degradation over time. The degradation rate was determined as the reduction of wet weight (*W*_*t*_) over time

4 $$de\mathrm{grad}ation\;ratio=\left(1-\frac{W_{t}}{W_{swollen}}\right)\times 100\%$$

where *W*_*t*_ is the wet weight at time *t*, and *W*_swollen_ is the initial weight after the preswelling phase.

The equilibrium phase is shown in the plots but is shaded to distinguish it from the enzymatic degradation phase. Each test was carried out with *n* = 5 or 6 replicates per formulation.

### 4.15 Measurements of Microgel Size and Interstitial Matrix Volume in oTHAm:THA Hydrogel Microparticle Composites by Confocal Microscopy

For confocal imaging, 0.1 wt % fluorescein isothiocyanate–dextran (FITC-Dextran, Sigma-Aldrich) with an average *M*_w_ of 2 MDa was added to either the oTHA microgels or THA interstitial matrix precursor solutions prior to photocross-linking. Labeled microgels in the PBS suspension and hydrogel microparticle composites with labeled interstitial matrices were imaged using an upright confocal microscope (LSM 800; Carl Zeiss AG) equipped with a 5x objective lens. A λ = 488 nm laser was used for imaging. Microgel suspensions obtained from the method described above were diluted 5 times in PBS after the centrifugation step (unpacked by vacuum) to yield a suspension that was imaged. In contrast, 175 μL of the hydrogel microparticle composite precross-linked solution was gently spread within a PDMS mold with wells of 15 mm diameter and 5 mm height. They were subsequently photocross-linked, resulting in an approximate hydrogel with a diameter of 7 mm and a height of 1 mm. These were then coated with a thin layer of PBS prior to fluorescence imaging. Ten individual single images and one volumetric Z-stack measuring a range of 60 μm with a *z*-spacing of 20 μm between successive stack slices were imaged across randomly selected regions of interest. For the hydrogel microparticle composites, five single images and one volumetric *Z*-stack were obtained. All images were taken at a 1024-pixel x 1024-pixel size. The size of the microgels was quantified using ImageJ by calculating the average surface area of the individual microgels identified in each image. To understand the particle shape better, circularity, which provides a measure of shape regularity in two dimensions (2D), was calculated according to the following equation

5 $$\mathrm{circularity}=\frac{4\pi \times \mathrm{area}}{{\left(\mathrm{perimeter}\right)}^{2}}$$

with values ranging from 0.00 to 1.00, where 1.00 indicates a perfect circle, while particles with a circularity below 0.5 are classified as highly irregular in shape, often characterized by jagged or nonuniform boundaries. The dark and bright areas of each image obtained for the hydrogel microparticle composite were calculated using ImageJ, where the intensity of pixels <40 was considered dark areas and ≥40 were considered bright areas. Between 8 and 10 images collected either from individual or *Z*-stacks were used for analysis.

### 4.16 Cell Culture of L929 Mouse Fibroblasts

L929 mouse fibroblast cells (Sigma-Aldrich, Cat. No. 85011425) were cultured in Dulbecco’s Modified Eagle’s Medium Low Glucose (DMEM LG; Gibco) supplemented with 10% (v/v) fetal bovine serum (FBS; Corning) and 1% (v/v) penicillin–streptomycin (100 U mL^–1^ penicillin and 100 μg mL^–1^ streptomycin; Gibco). The cells were maintained at 37 °C in a humidified incubator with 5% CO_2_. The culture medium was refreshed every 2 days, and subculturing was performed when the cells reached approximately 80% confluency using standard trypsin–EDTA detachment procedures. Cells at passage 16 were used for all subsequent experimental analyses.

### 4.17 Metabolic Activity of L929 Cells

Metabolic activity, reflecting cell viability, was quantified using the CellTiter-Blue assay (Promega, Switzerland). All tested precross-linked formulations (2 w/v% polymer content, 0.1 mM Ru, and 5 mM SPS) were first prepared in sterile conditions, with prior UV sterilization of the as-synthesized powder, followed by preparation using all 0.22 μm filtered solutions (PBS, Ru, and SPS). One milliliter of each formulation (per repeat) was then gently pipetted into 24-well plates (TPP), and all formulations were cross-linked following the photocross-linking method described above. Subsequently, all of them were incubated with 1 mL of L929 cell culture medium for 24 h at 37 °C. The cell culture medium alone was incubated without any gel under identical conditions to serve as a control. L929 cells were seeded on 96-well plates at 4000 cells cm^–2^ and left for 24 h for attachment. After 24 h, the conditioned cell culture medium was extracted from the hydrogels and used to change the medium of L929 cells. L929 cells were further incubated for 24 h with conditioned cell culture medium and analyzed for their metabolic activity following the manufacturer’s instructions. A 1:5 (v/v) CellTiter-Blue reagent-to-medium mixture was added to each well, and after 2 h of incubation, fluorescence was measured at 560 nm excitation and 590 nm emission after transferring the reagent–media mixture to a 96-well plate using a spectrophotometer (Infinite 200 Pro, Tecan, Switzerland). Medium alone served as a blank to subtract background fluorescence.

### 4.18 L929 DNA Content Measurement

The cellular DNA content was quantified by using the Hoechst 33258 fluorescent dye assay. Following the metabolic activity experiments, the cells were washed with PBS and digested overnight at 56 °C with 60 μL of proteinase K to ensure complete cell lysis. The enzymatic activity was subsequently inactivated by heating the samples at 95 °C for 10 min. The lysates were stored at −20 °C until further analysis. For measurement, 40 μL of the samples were mixed with 160 μL Hoechst 33258 dye solution (Sigma-Aldrich). Fluorescence intensity was recorded by using a microplate reader at 360 nm excitation and 465 nm emission (Infinite 200 Pro, Tecan, Switzerland). DNA concentration was calculated from a standard curve generated with calf thymus DNA, and results were expressed as micrograms of DNA per sample.

### 4.19 Cytotoxicity of L929 Cells Using Lactate Dehydrogenase (LDH) Assay

Cell cytotoxicity was assessed using a lactate dehydrogenase (LDH) Cytotoxicity Detection KitPLUS (Roche Diagnostics, Cat. No. 04744926001), which quantifies the LDH released from damaged cells into the culture medium. Culture supernatants were collected from the cells prepared for the metabolic activity experiment and stored at 4 °C until analysis. For measurement, 100 μL of the sample supernatant was mixed with an equal volume of freshly prepared reaction mixture according to the manufacturer’s instructions and incubated for 30 min at room temperature, protected from light. The reaction was terminated by adding 50 μL of stop solution, and the absorbance was measured at 490 nm with a reference wavelength of 600 nm by using a microplate reader (Infinite 200 Pro, Tecan, Switzerland). Cells treated with lysis buffer containing Triton X-100 served as the positive control (100% LDH release), while untreated cells served as the negative control. The results were expressed as the percentage of LDH release relative to that of the positive control, indicating the degree of cytotoxicity.

### 4.20 Generation of Ring-Shaped Cartilage Explants

Bovine stifle joints were obtained from a local abattoir (Metzgerei Angst AG, Zurich, CH) 48 h after slaughter. Osteochondral explants were harvested from the patellar groove, as previously described.^85^ Briefly, cylindrical osteochondral explants were prepared using a trephine drill (Peertools AG, Ftan, Switzerland) with an inner diameter of 8 mm connected to a Bosch compact drill press and rinsed in PBS containing 100 U mL^–1^ penicillin and 100 μg mL^–1^ streptomycin (1% P/S, Gibco). Subsequently, full-thickness cartilage (∼2 mm) was carefully separated from the subchondral bone at the cartilage–bone interface. Using a custom-designed metallic ring-forming device (Figure S18) and a 4 mm biopsy punch (Kai Medical), cartilage rings with a 4 mm central hole were generated for subsequent push-out assays. The cartilage rings were cultured overnight in high-glucose Dulbecco’s Modified Eagle’s Medium (DMEM, 4.5 g L^–1^ glucose, Gibco), supplemented with 10% fetal bovine serum (FBS, Corning), 100 U mL^–1^ penicillin, and 1% P/S at 37 °C and 5% CO_2_.

### 4.21 Push-Out Adhesion Test

The push-out method for adhesion measurements was readapted from Behrendt et al.^68^ First, to determine the adhesion of the hydrogel formulations, cartilage rings were prepared as described above. On the measurement day, they were filled with hydrogel precursor formulations prepared as described above. The rings were then individually laid out in a 12-well plate, rinsed in PBS, and blotted. Subsequently, for each ring, a volume of 27–30 μL was gently pipetted in the dark. Photocross-linking was then done the same as described above for the case in the molds, *i.e*., for three minutes on each side under LED irradiation with λ = 450 nm and a power of 5 mW cm^–2^. The cartilage laden with hydrogel was subsequently immersed in 1 mL of PBS and set aside for a couple of minutes at room temperature before measurements. The samples were always prepared fresh on the day of measurement. After gelation at room temperature, a push-out test was performed with an Instron 5866 electromechanical test device equipped with a 10 N load cell at a speed of 0.5 mm min^–1^. Five cartilage rings were used for each condition, with cartilage rings isolated from at least 2 different animals for each group tested. The bonding strength (σ, Pa) was calculated by dividing the measured load peak (N) of each sample by the bond surface area (m^2^).

### 4.22 Viability Assay of Cartilage Rings

To assess the cell viability of the cartilage rings, live and dead staining was performed after an overnight culture. The cartilage rings were incubated in PBS containing 4 μM calcein AM (Sigma-Aldrich) and 2 μM ethidium homodimer (EthD, Sigma-Aldrich) for 1 h at 37 °C. The cartilage rings were then rinsed with PBS, and imaging was performed immediately using confocal microscopy (LSM 800; Carl Zeiss AG).

### 4.23 Statistical Analysis

All results were analyzed and presented using GraphPad Prism 10 (v10.3.2, GraphPad Software Inc.). The results are shown as the mean ± standard deviation (SD), with individual points marked. To determine statistical significance, one-way analysis of variance (ANOVA) with Šídák’s multiple comparisons or Mann–Whitney multiple comparisons were used and noted in each case in the figure description. Statistically significant results were considered for *p* < 0.05 (* *p* < 0.05, ** *p* < 0.01, *** *p* < 0.005, and **** *p* < 0.001). Lack of significance was indicated as ns when *p* ≥ 0.05.

## Supplementary Material



## Data Availability

All research data supporting this publication are directly available within this publication and associated Supporting Information.

## Notes

Spectroscopic (NMR/FTIR) and viscosity characterization of the oTHA batches from all reactions as compared to THA (Section S1); dynamic and static light scattering/molecular mass measurements and comparison to SEC-SALS (Section S2); supporting data for comparison of single-component hydrogels made of THA versus oTHA (Section S3); supporting data for combining oTHA and THA in a two-component blended hydrogel formulation section (Section S4); supporting data for cartilage ring development and push-out-test adhesion testing (Section S5); microgel characterization (Section S6); supporting data for combining oTHA microgels within THA matrix–hydrogel microparticle composites approach (Section S7) (PDF)
