# Supplementary material for: Blended and Microparticle Composite Hyaluronan Hydrogels with Programmable Degradation through Selective Oxidation
Source: ACS Polym Au. 2025 Dec 17;6(1):226–45. doi: 10.1021/acspolymersau.5c00129 (PMC12903431; doi:10.1021/acspolymersau.5c00129)
Supplement: Supplementary file 1 [file lg5c00129_si_001.pdf]

Blended and microparticles composite hyaluronan hydrogels with programmable degradation through selective oxidation – Supporting Information

Melanie Grimm[1,2], Fiona Ye Rojo Acero[1], Fatemeh Safari[1], Desiré Venegas-Bustos[3], Andreas Wagner,[4] Clara Presciutti[1], Wen Chen[1], Matteo D'Este[1*] and Jacek K. Wychowaniec[1,*]

[1] AO Research Institute Davos, Clavadelerstrasse 8, Davos, 7270, Switzerland

[2] ETH Zürich, Rämistrasse 101, Zürich 8092, Switzerland

[3] Bioforge Lab, LaDIS, CIBER-BBN, Edificio LUCIA, Universidad de Valladolid, Valladolid, 47011, Spain

[4]Anton Paar GmbH, Anton-Paar-Str. 20, 8054 Graz, Austria

*Corresponding authors

M.D. e-mail: matteo.deste@aofoundation.org

J.K.W. e-mail: jacek.wychowaniec@aofoundation.org

Outline:

Section 1. Spectroscopic (NMR/FTIR) and viscosity characterisation of the oTHA batches from all reactions as compared to THA.

Section 2. Dynamic light scattering / molecular mass measurements and comparison to SEC-SALS.

Section 3. Supporting data for comparison of single component hydrogels made of THA versus oTHA.

Section 4. Supporting data for combining oTHA and THA in a two-component blended hydrogel formulation section.

Section 5. Supporting data for cartilage ring development and push-out-test adhesion testing.

Section 6. Microgels characterization.

Section 7. Supporting data for combining oTHA microgels within THA matrix – hydrogel microparticles composites.

Section 1. Spectroscopic (NMR/FTIR) and viscosity characterisation of the oTHA batches from all reactions as compared to THA.

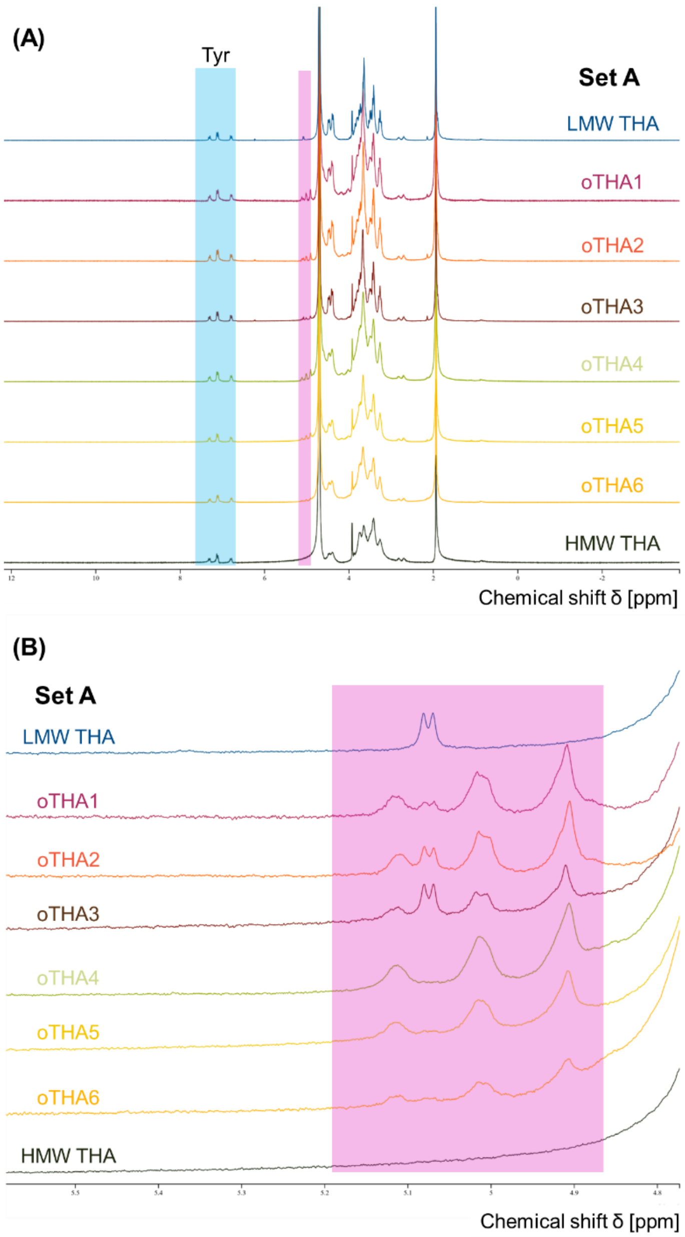

Figure S1. *^{1}H NMR spectra of Set A of reactions (see* ***Table 1*** *in main text), including LMW THA, oTHA1, oTHA2, oTHA3, oTHA4, oTHA5, oTHA6 and HMW THA. The highlighted blue region contains the resonances at 7.17–7.19 ppm, 6.85-6.86 ppm, and 7.36-7.38, and is characteristic for the tyramine functionalization of HA conforming to the previously published spectra.*[1, 2] ***(A)*** *Depicts a wider view from 0 to 12 ppm, whereas* ***(B)*** *shows a zoom into the 4.8 – 5.5 ppm region (with peaks at 4.9, 5.0 and 5.12 ppm) and showing changes due to aldehyde incorporation.*

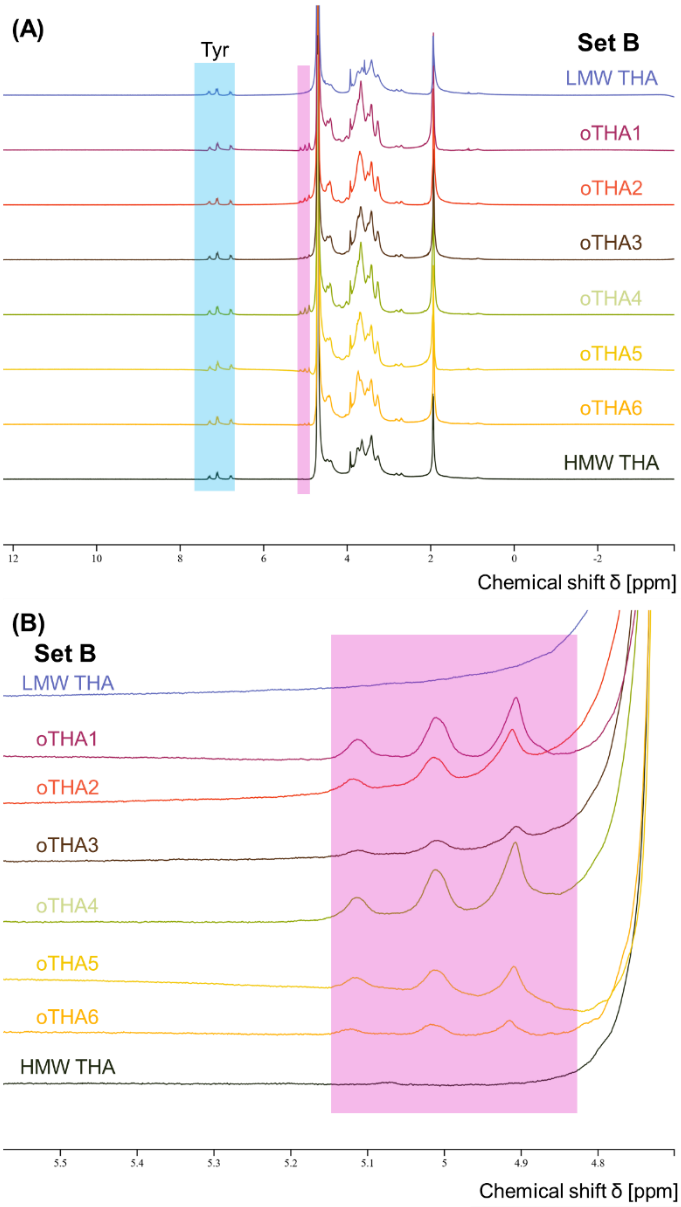

Figure S2. *^{1}H NMR spectra of Set B of reactions (see* ***Table 1*** *in main text), including LMW THA, oTHA1, oTHA2, oTHA3, oTHA4, oTHA5, oTHA6 and HMW THA. The highlighted blue region contains the resonances at 7.17–7.19 ppm, 6.85-6.86 ppm, and 7.36-7.38, and is characteristic for the tyramine functionalization of HA conforming to the previously published spectra.[1, 2]* ***(A)*** *Depicts a wider view from 0 to 12 ppm, whereas* ***(B)*** *shows a zoom into the 4.8 – 5.5 ppm region (with peaks at 4.9, 5.0 and 5.12 ppm) and showing changes due to aldehyde incorporation.*

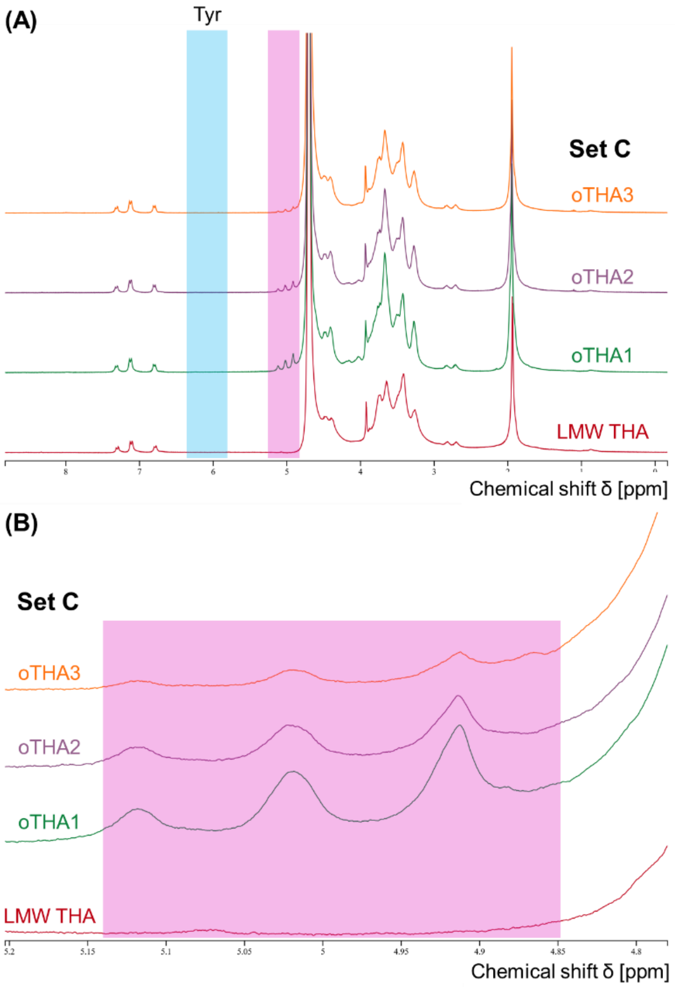

Figure S3. *^{1}H NMR spectra of Set B of reactions (see* ***Table 1*** *in main text), including LMW THA, oTHA1, oTHA2, oTHA3, oTHA4, oTHA5, oTHA6 and HMW THA. The highlighted blue region contains the resonances at 7.17–7.19 ppm, 6.85-6.86 ppm, and 7.36-7.38, and is characteristic for the tyramine functionalization of HA conforming to the previously published spectra.[1, 2]* ***(A)*** *Depicts a wider view from 0 to 12 ppm, whereas* ***(B)*** *shows a zoom into the 4.8 – 5.5 ppm region (with peaks at 4.9, 5.0 and 5.12 ppm) and showing changes due to aldehyde incorporation.*

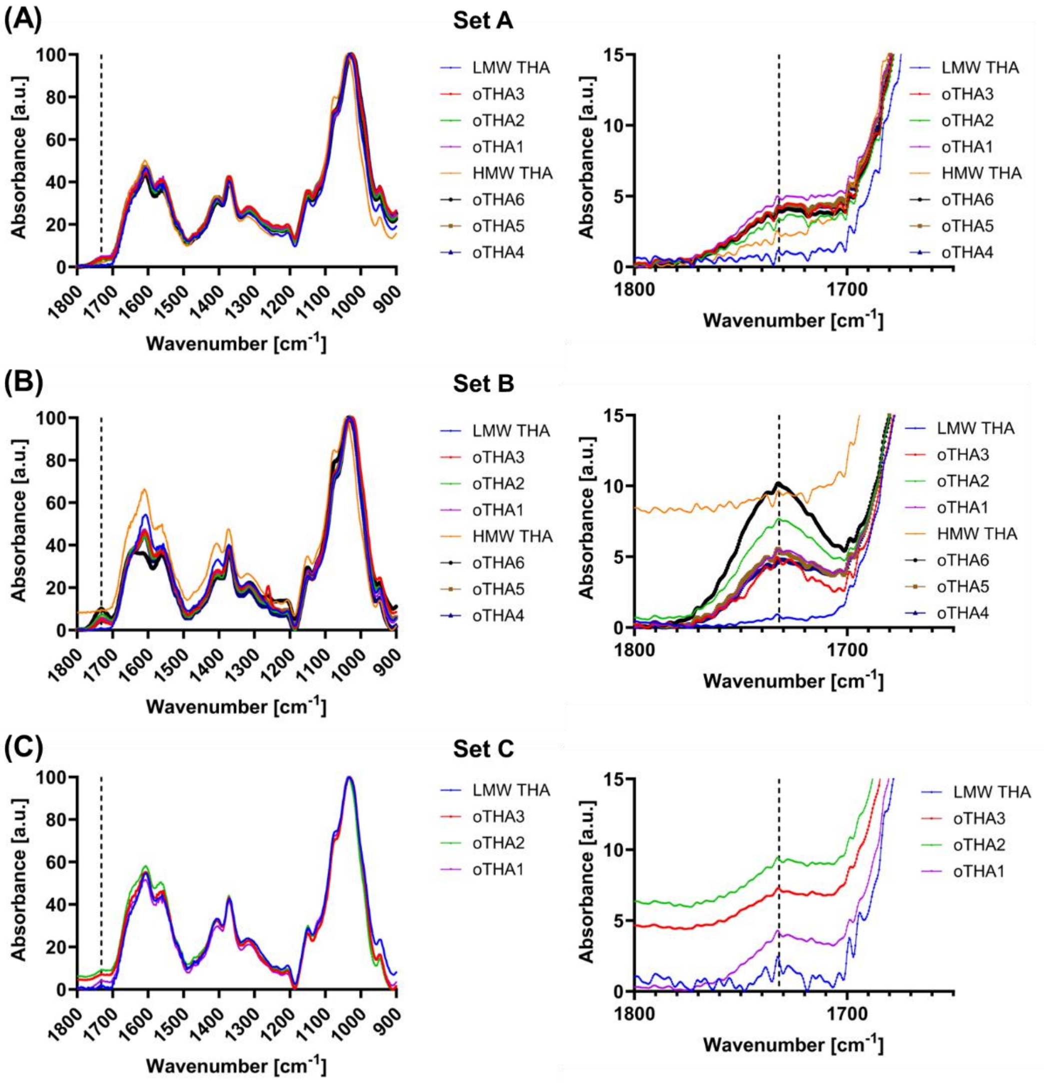

Figure S4. *ATR-FTIR spectra obtained for all polymers from* ***(A)*** *set A,* ***(B)*** *set B, and* ***(C)*** *set C, of reactions (see* ***Table 1*** *in main text), including LMW THA, oTHA1, oTHA2, oTHA3, oTHA4, oTHA5, oTHA6 and HMW THA. For each full spectra, a zoom into the aldehyde region is provided on the right-hand side (from 1650 to 1800 cm^{-1}). Vertical dashed line indicates the position of the characteristic band for aldehyde peak at 1732 cm^{-1}. The HMW THA in Set B had a slight offset of the baseline as compared to the other measurements, however we did not attempt to correct this as the lack of aldehyde peak is clearly visible in this case. Set C did not include HMW THA syntheses, as explained in the main text.*

Table S1. *Ratios of FTIR aldehyde peak (1730 cm^{-1}) to other functional group peaks in three independent oTHA sets of syntheses (Set A, Set B and Set C). Blue colour highlights the values for oTHA samples, whereas grey remains for non-modified THA samples.*

**Sample**	**LMW THA**	**oTHA3**	**oTHA2**	**oTHA1**	**HMW THA**	**oTHA6**	**oTHA5**	**oTHA4**
**Ratio [%]**	**Set A**							
Aldehyde vs C-O-C stretching I_{1732}/I_{1610}	2.45	10.20	8.34	11.14	6.52	9.49	9.32	9.51
Aldehyde vs amide II I_{1732}/I_{1557}	2.90	10.91	9.73	11.86	8.36	11.28	10.83	10.77
Aldehyde vs C-H bending I_{1732}/I_{1370}	2.81	10.50	9.01	11.86	8.31	10.31	10.41	10.39
Aldehyde vs C-O stretching I_{1732}/I_{1030}	1.14	4.49	3.71	5.04	3.28	4.10	4.39	4.33
	**Set B**							
Aldehyde vs C-O-C stretching I_{1732}/I_{1610}	1.73	10.66	17.59	11.58	*	28.07	12.25	10.53
Aldehyde vs amide II I_{1732}/I_{1557}	2.32	12.81	20.14	14.94	*	28.25	14.59	13.14
Aldehyde vs C-H bending I_{1732}/I_{1370}	2.34	13.16	20.24	14.57	*	25.96	14.23	12.87
Aldehyde vs C-O stretching I_{1732}/I_{1030}	0.94	4.98	7.70	5.49	*	10.19	5.56	4.78
	**Set C**							
Aldehyde vs C-O-C stretching I_{1732}/I_{1610}	4.48	13.19	16.31	8.30	-	-	-	-
Aldehyde vs amide II I_{1732}/I_{1557}	5.47	15.69	18.72	9.76	-	-	-	-
Aldehyde vs C-H bending I_{1732}/I_{1370}	5.70	16.83	21.39	10.31	-	-	-	-
Aldehyde vs C-O stretching I_{1732}/I_{1030}	2.44	7.28	9.47	4.27	-	-	-	-

** HMW THA in Set B had uneven background for all repeats causing the issue with ratio calculations which came unrealistic as compared to other syntheses and measurements.*

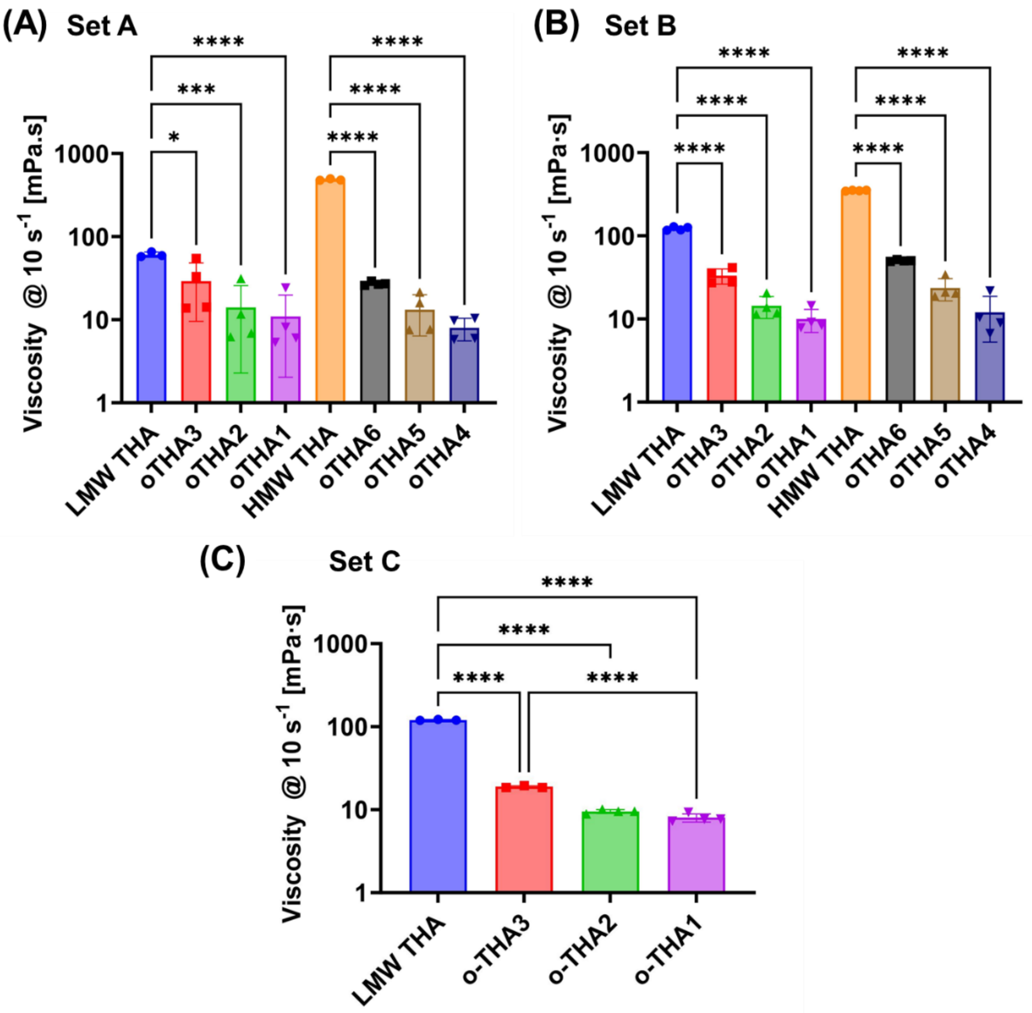

Figure S5. *Viscosity, η, extracted from flow curve measurements, at shear rate, $\dot{\gamma}$ = 10 s^{-1} and temperature 23 °C, all polymers from* ***(A)*** *Set A,* ***(B)*** *Set B, and* ***(C)*** *Set C, of reactions (see* ***Table 1*** *in main text), including LMW THA, oTHA1, oTHA2, oTHA3, oTHA4, oTHA5, oTHA6 and HMW THA. Statistical analysis was done by one-way analysis of variance (ANOVA) with Šídák's multiple comparisons. A statistically significant results were considered for p<0.05 (* - <0.05, ** - <0.01, *** - <0.005 and **** - <0.001).*

Section 2. Dynamic light scattering / molecular mass measurements and comparison to SEC-SALS

In first instance, we ran SEC-MALS measurement on polymer from set A syntheses. SEC-MALS provides absolute M_w and size values without reference to external relative calibrations. All set A samples were completely soluble in 0.2 M NaCl solvent without evident aggregation, presence of gel or any insoluble particles. In general, the SEC fractionation of all samples was considered efficient and allowed for complete determination of macromolecular M_w distribution, M_w averages, and polydispersity indexes. The obtained values confirmed significant fragmentation of the chains, which, was more pronounced for the set of HMW samples, as compared to the LMW (***Table S2***). M_w average of LMW THA sample decreased by 24% from the nominal value of 280 kDa, while HMW THA showed a 75% reduction of M_w from the nominal value of 1.64 MDa HMW HA, indicating that tyramination synthesis itself affects the M_w (***Table S2***). The degradation of HA via decrease of its M_w has been noted before under a range of mechanical stimuli,[3, 4] pointing to an increased dissolution time under stirring for HMW HA as a plausible key aspect responsible for this reduction.

To complement the SEC-MALS results, we performed molecular mass measurements using DLS/SLS, which, although less accurate, is more readily available for most laboratories. For control LMW (***Figure S6***) and HMW HA (***Figure S7***), we observed a single population across the studied concentrations, without significant aggregation, except at 2 mg mL^{-1}, indicating samples exhibited measurable conditions for molecular mass measurement. Similarly, for LMW THA (***Figure S8*** and ***Figure S9***) and HMW THA (***Figure S10***), the monodisperse populations were detected by DLS until higher concentrations (> 1 mg mL^{-1}), allowing molecular mass measurement for these samples. No aggregation was observed particularly for HMW THA (***Figure S10***); however, it did not prevent molecular mass determination. For oTHA1 (***Figure S11***) and oTHA4 (***Figure S12***) in PBS, strong aggregation was evident across the full concentration range tested, precluding reliable molecular mass determination. Although molecular mass measurements were attempted for all samples (oTHA1–oTHA6), all yielded negative second virial coefficients in the Debye plots, indicating attractive

interactions and solvent-driven aggregation. Aggregation typically results in nonlinearity in the plot of $K{\cdot}c/R(\theta)$ versus concentration c, disrupting the assumption of monodispersity necessary for accurate molar mass extrapolation to zero concentration. Consequently, under these conditions, the molecular mass measurements proved unreliable and were excluded from further analysis. As noted in our DLS measurements, significant aggregation occurred at the very low concentrations, with particle size consistently increasing to the 200-600 nm range (***Figure S11*** and ***Figure S12***). We hypothesise that oxidation reduced chain length and disrupted polymer conformation, increasing flexibility and promoting chain-chain association. In addition, aldehyde groups likely disturbed the native hydration shell, partially neutralizing charges and promoting electrostatic screening and aggregation in the high ionic strength environment of PBS.[5] The reduced charge density (due to aldehyde formation) combined with this screening effect may have led to suboptimal solubility and promoted the observed aggregation.

SLS measurements yielded molecular weights of 177 kDa and 1.24 MDa, for unmodified LMW and HMW HA, respectively (***Table S2***). These values are in line with the specified ranges of 150 – 350 kDa (average M_w 280 kDa) for LMW HA, and >1.0 MDa (average M_w = 1.64 MDa) for HMW HA, provided in the certification from the supplier. The values obtained for LMW THA in both set A and set B fell also within the original certification of analysis (131 and 247 kDa), whereas for HMW THA the values were 361 and 353 kDa. These results were in line with the obtained SEC-MALS values, indicating ~75% decrease from the original M_w of native HMW HA used for syntheses. We acknowledge the limitations of DLS/SLS measurements, which may exhibit high variability, primarily due to any modified HA polymer chains reacting strongly to environmental conditions in solution (pH, temperature, ionic strength, concentration, etc.).[6-8]

Table S2. *SEC-MALS and DLS molecular mass measurements depicting average* M_w *for two independent sets of oTHA syntheses (**Set A** and **Set B**). Green values indicate trusted and validated measurements. Aggregating indicates that the 2nd viral coefficient was negative in molecular mass measurements and/or that consistent multiple populations were detected in standard DLS measurements across all tested concentration series, as depicted exemplary for oTHA1 and oTHA4 from Set B in **Figure S11** and **Figure S12** below, respectively. SEC-MALS measurements were performed on samples dissolved in 0.2 M NaCl, whereas DLS was performed on samples dissolved in PBS. In main text we note* that t*he solvent may significantly influence solubility and aggregation behaviour of the samples and therefore the outcome of the measurement.*

Sample	$NaIO_4$:THA	Set A		Set B
		SEC-MALS	DLS	DLS
		Average M_w [kDa]		
**LMW HA**	**-**	285*	177	177
**LMW THA**	**-**	213	131	247
**oTHA3**	**1:3**	80	Aggregating	Aggregating
**oTHA2**	**2:3**	56	Aggregating	Aggregating
**oTHA1**	**1:1**	50	Aggregating	Aggregating
**HMW HA**	**-**	1600	1240&	1240&
**HMW THA**	**-**	406	361	353
**oTHA6**	**1:3**	38	Aggregating	Aggregating
**oTHA5**	**2:3**	19	Aggregating	Aggregating
**oTHA4**	**1:1**	20	Aggregating	Aggregating

**The value for LMW HA was not measured but taken from the original certification report from the Contipro Nutrihyl company.*

& Although the measurements were internally consistent, none of the DLS values produced molecular mass calculations that aligned with literature data. Consequently, literature values were employed for calculating the molecular mass of HMW HA.[6]

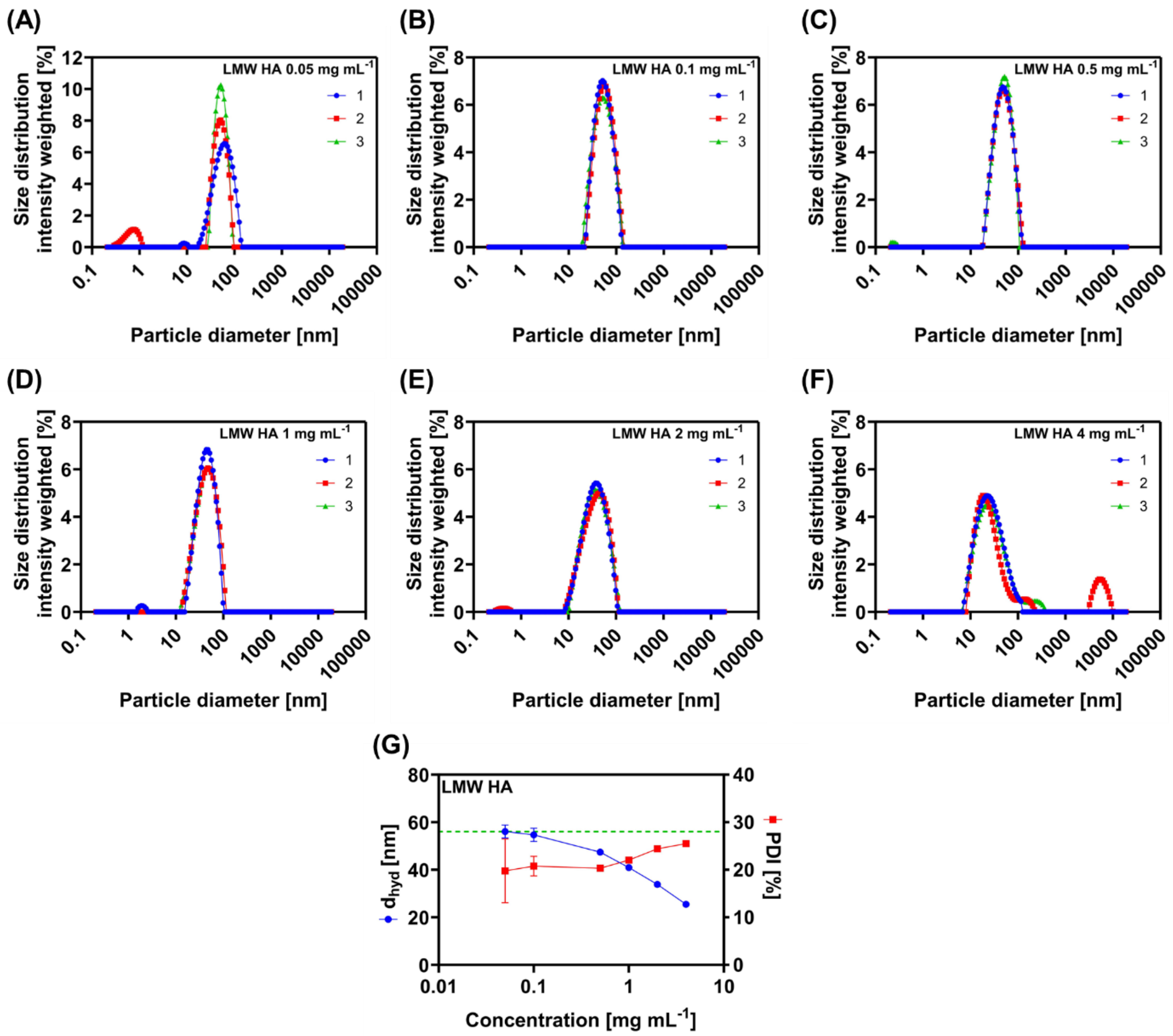

Figure S6. *DLS intensity weighted size distribution of LMW HA as a function of noted concentration ranging from **(A)** 0.05 mg mL^{-1} to **(F)** 4 mg mL^{-1}. 3 repeats are shown in different colours for each concentration. **(G)** Extracted hydrodynamic size (d_{hyd}) and polydispersity index (PDI) from the DLS measurements plotted as a function of concentration. Green dashed line depicts the d_{hyd} value used for coil shape correction factor in molecular mass measurement.*

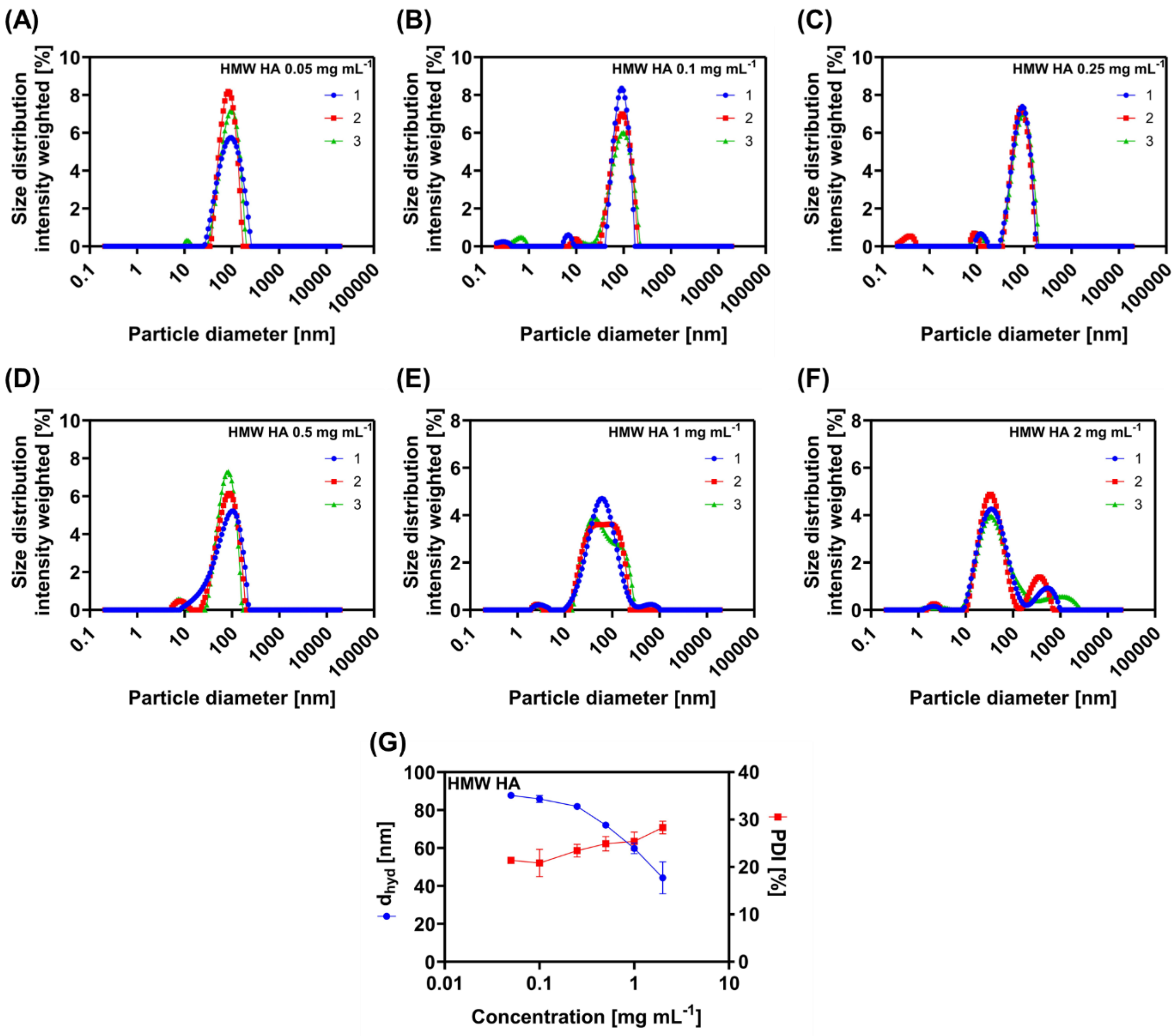

Figure S7. *DLS intensity weighted size distribution of HMW HA as a function of noted concentration ranging from* ***(A)*** *0.05 mg mL^{-1} to* ***(F)*** *2 mg mL^{-1}. 3 repeats are shown in different colours for each concentration.* ***(G)*** *Extracted hydrodynamic size (d_{hyd}) and polydispersity index (PDI) from the DLS measurements plotted as a function of concentration.*

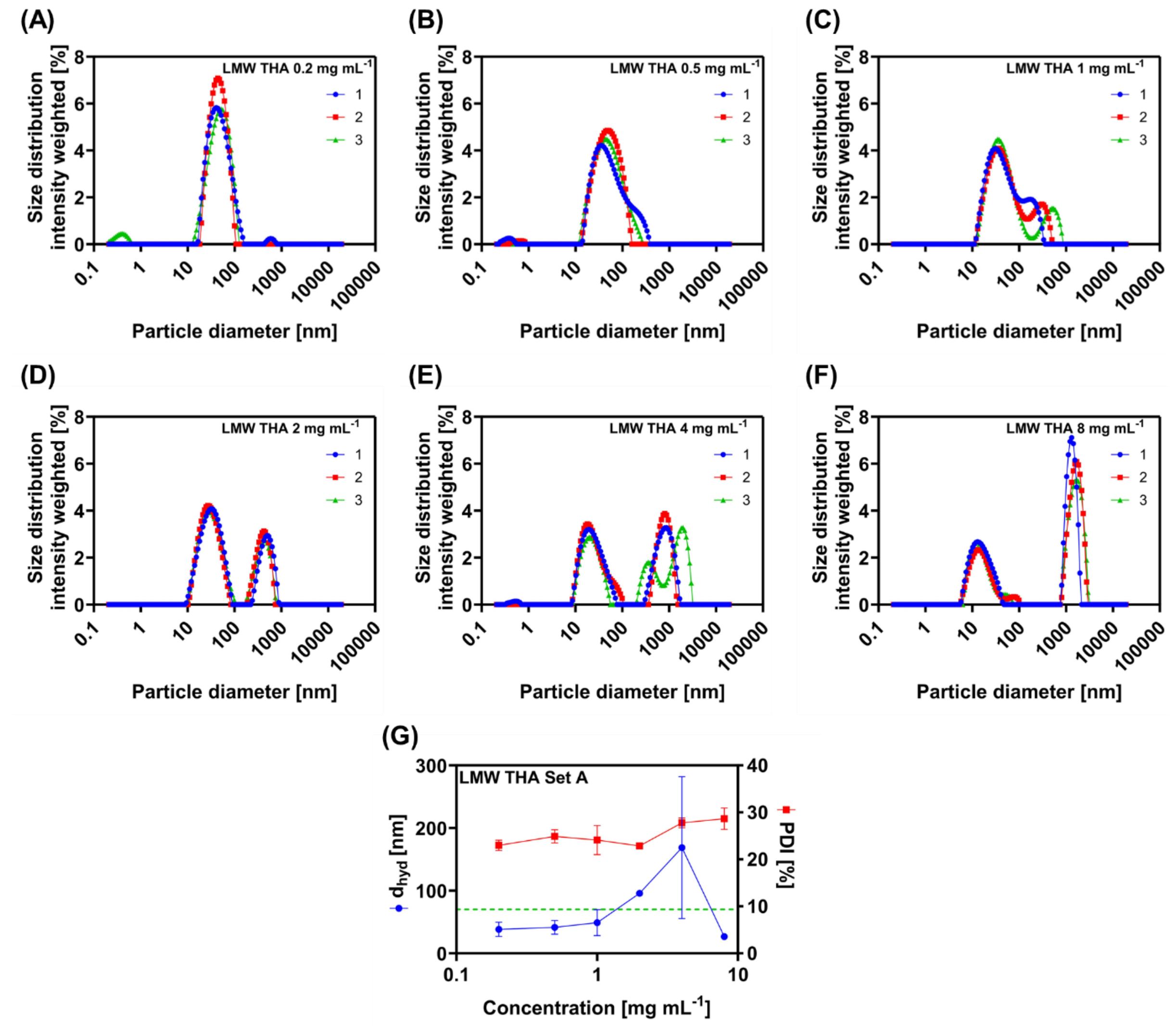

Figure S8. *DLS intensity weighted size distribution of LMW THA from Set A as a function of noted concentration ranging from* ***(A)*** *0.2 mg mL*$^{-1}$ *to* ***(F)*** *8 mg mL*$^{-1}$*. 3 repeats are shown in different colours for each concentration.* ***(G)*** *Extracted hydrodynamic size (*d_{hyd}*) and polydispersity index (PDI) from the DLS measurements plotted as a function of concentration. Green dashed line depicts the* d_{hyd} *value used for coil shape correction factor in molecular mass measurement.*

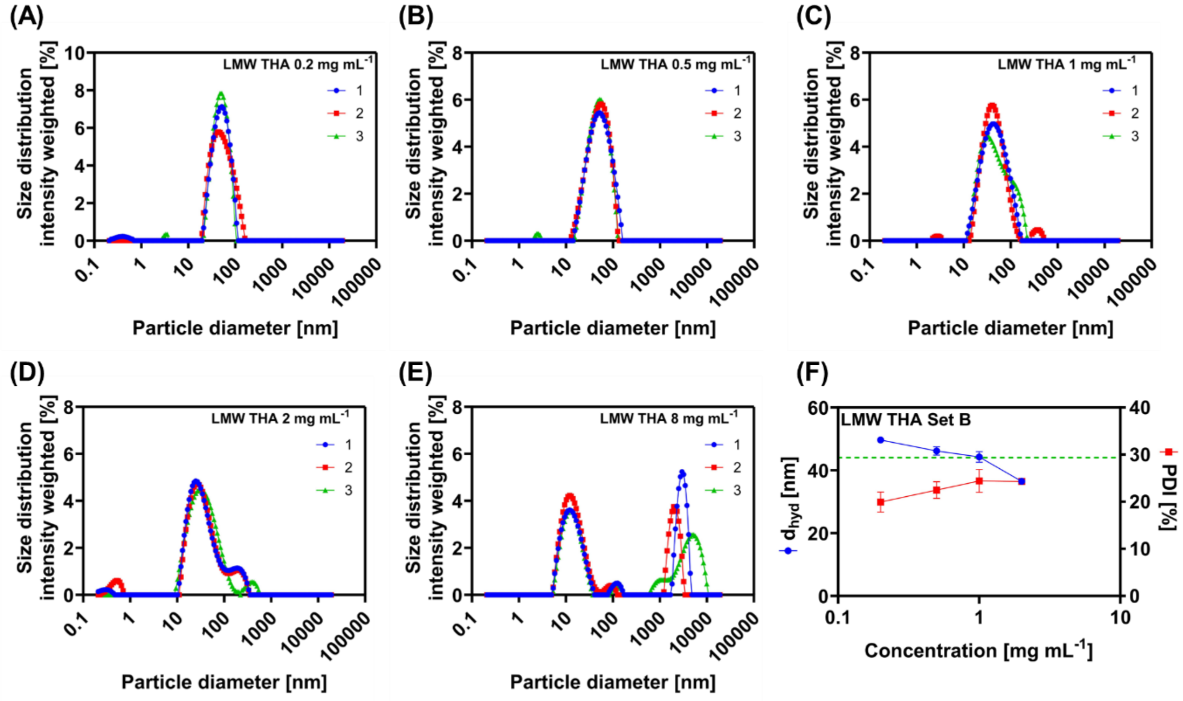

Figure S9. *DLS intensity weighted size distribution of LMW THA from Set B as a function of noted concentration ranging from* ***(A)*** *0.2 mg mL*$^{-1}$ *to* ***(E)*** *8 mg mL*$^{-1}$*. 3 repeats are shown in different colours for each concentration.* ***(F)*** *Extracted hydrodynamic size (*d_{hyd}*) and polydispersity index (PDI) from the DLS measurements plotted as a function of concentration. Green dashed line depicts the* d_{hyd} *value used for coil shape correction factor in molecular mass measurement.*

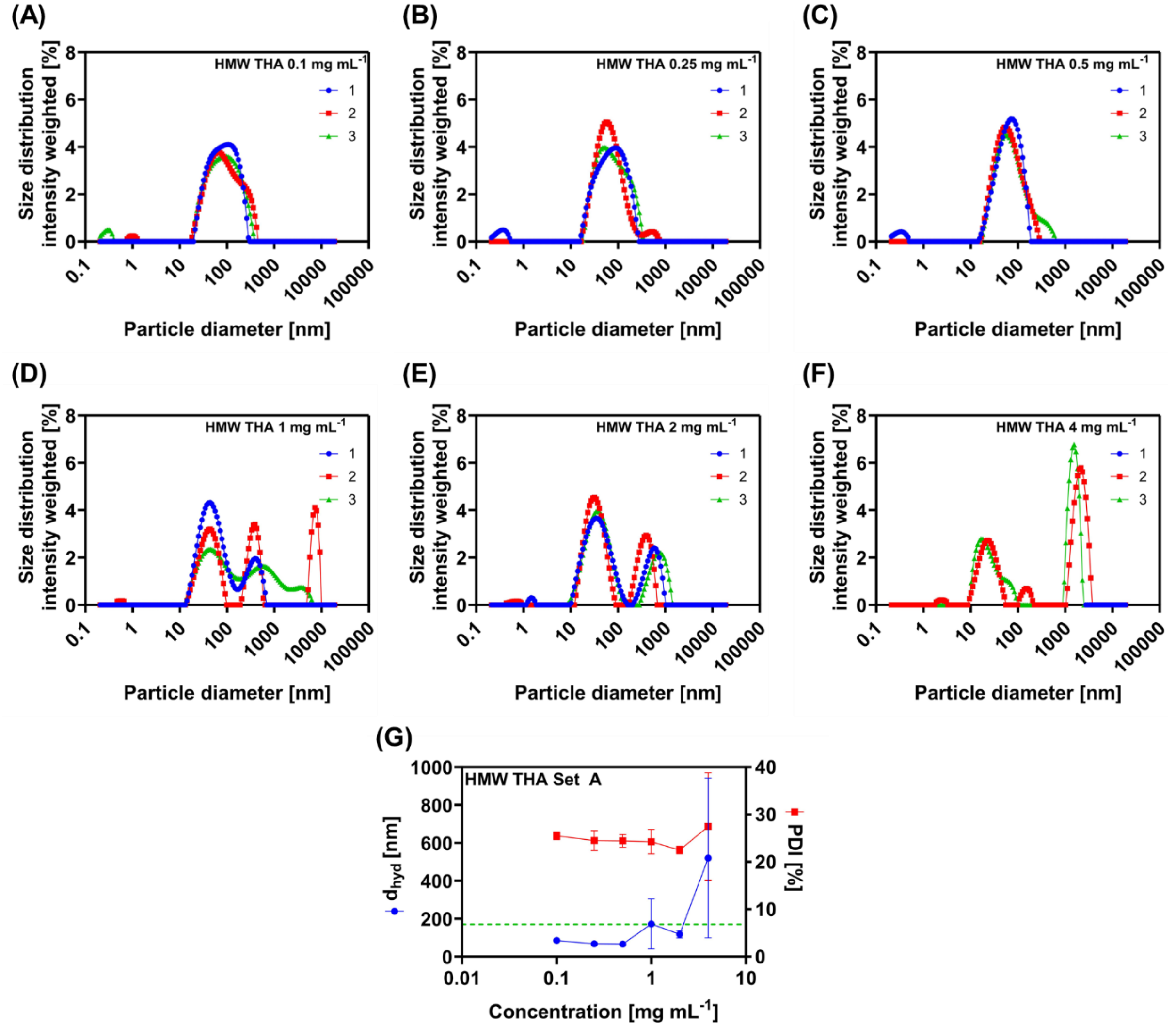

Figure S10. *DLS intensity weighted size distribution of HMW THA from Set A as a function of noted concentration ranging from* ***(A)*** *0.1 mg mL^{-1} to* ***(F)*** *4 mg mL^{-1}. 3 repeats are shown in different colours for each concentration.* ***(G)*** *Extracted hydrodynamic size (d_{hyd}) and polydispersity index (PDI) from the DLS measurements plotted as a function of concentration. Green dashed line depicts the d_{hyd} value used for coil shape correction factor in molecular mass measurement.*

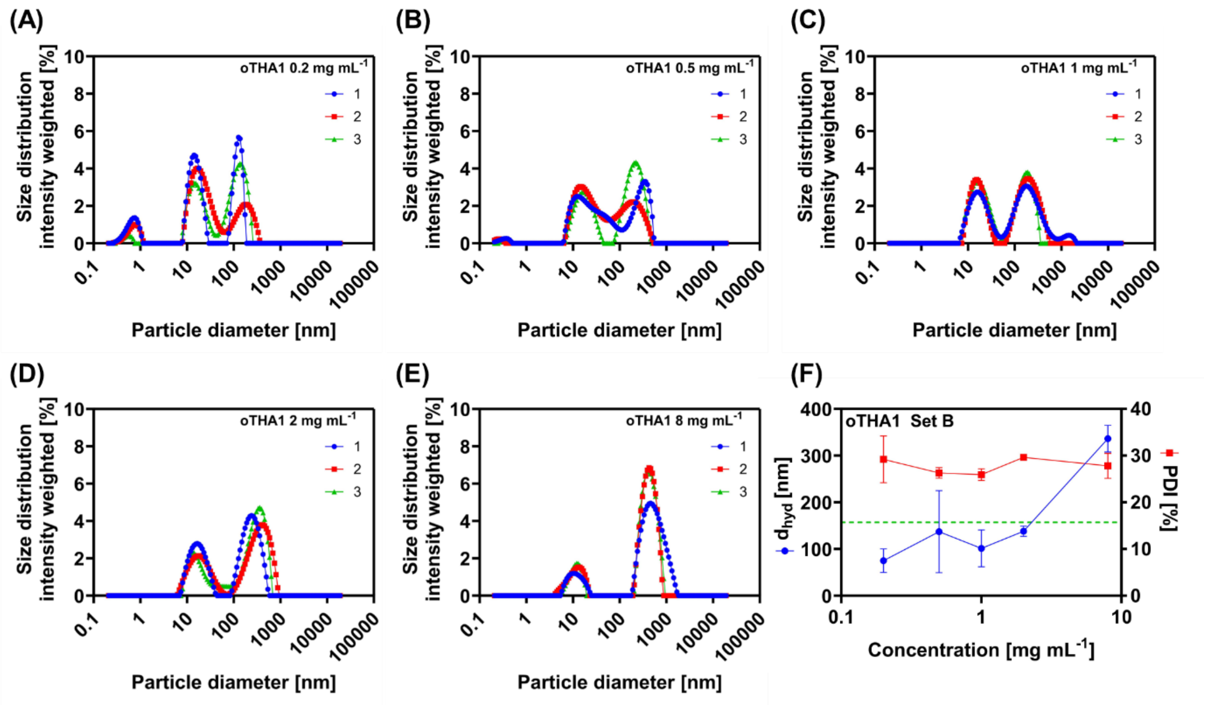

Figure S11. *DLS intensity weighted size distribution of oTHA1 from Set B as a function of noted concentration ranging from* ***(A)*** *0.2 mg mL^{-1} to* ***(E)*** *8 mg mL^{-1}. 3 repeats are shown in different colours for each concentration.* ***(F)*** *Extracted hydrodynamic size (d_{hyd}) and polydispersity index (PDI) from the DLS measurements plotted as a function of concentration. Green dashed line depicts the average d_{hyd} across all concentration measurements.*

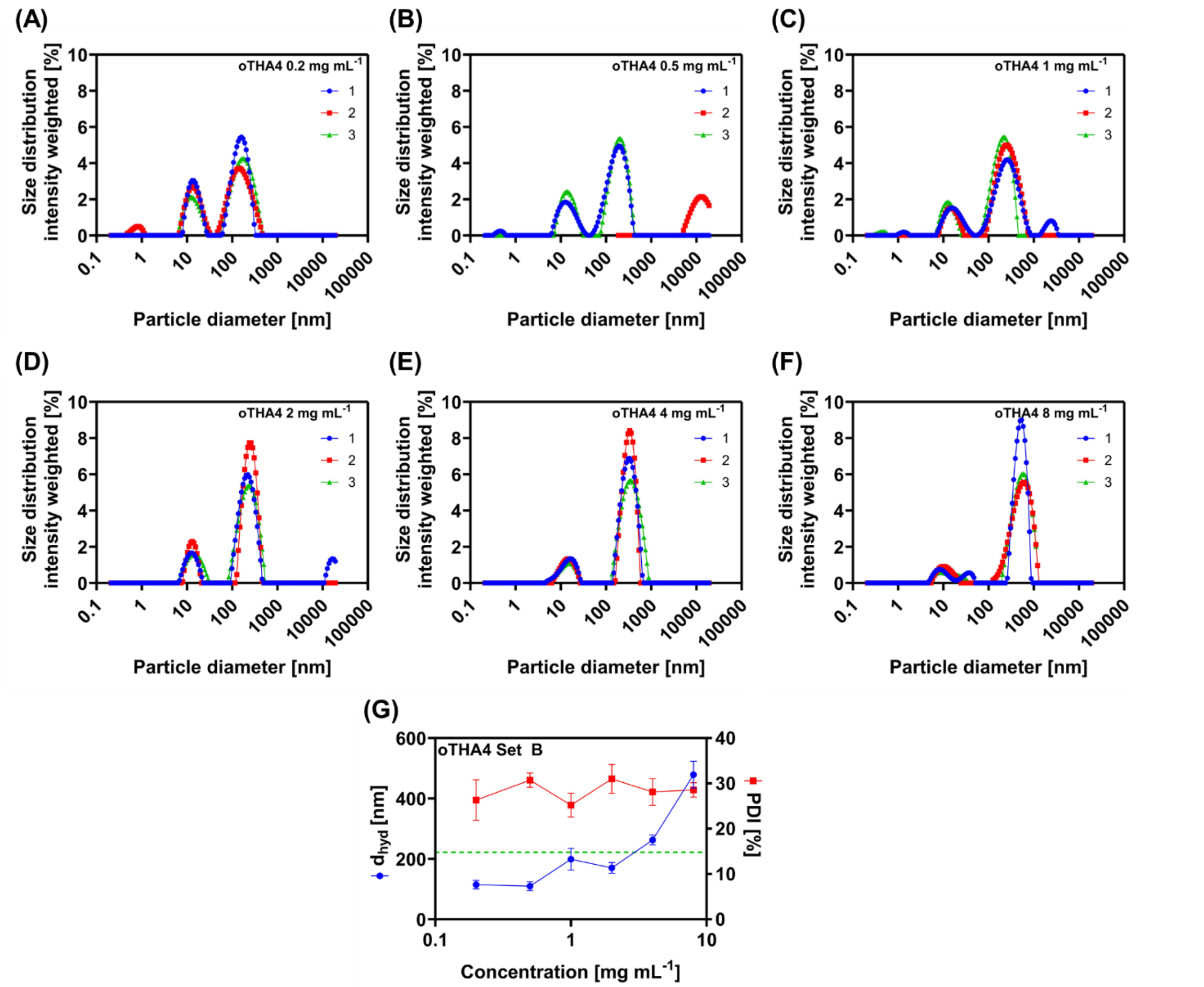

Figure S12. *DLS intensity weighted size distribution of oTHA4 from Set B as a function of noted concentration ranging from* ***(A)*** *0.2 mg mL*$^{-1}$ *to* ***(F)*** *8 mg mL*$^{-1}$*. 3 repeats are shown in different colours for each concentration.* ***(G)*** *Extracted hydrodynamic size (*d_{hyd}*) and polydispersity index (PDI) from the DLS measurements plotted as a function of concentration. Green dashed line depicts the average* d_{hyd} *across all concentration measurements.*

Section 3. Supporting data for comparison of single component hydrogels made of THA versus oTHA.

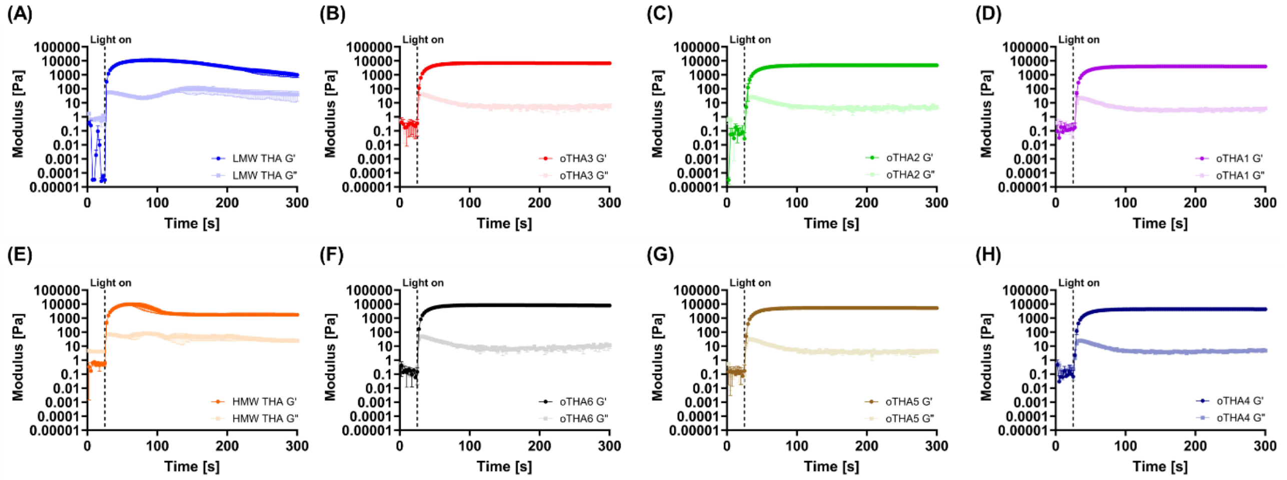

Figure S13. *Gelation kinetics of **(A)** LMW THA, **(B)** oTHA3, **(C)** oTHA2, **(D)** oTHA1, **(E)** HMW THA **(F)** oTHA6, **(G)** oTHA5, **(H)** oTHA4, 2 w/v% hydrogels (with 0.1 mM Ruthenium and 5 mM SPS) from **Set A** of reactions, at 23 °C. Average and standard deviation value is depicted in the graphs with n = 3 or 4 replicates of measurements. In most cases, the standard deviation error bars are smaller than data marks. The in-situ gelation process was measured on the quartz crystal stage by illuminating the bottom of the deposited sample with a full spectrum light as described in methods, and the light was switched on as indicated by the vertical dashed line in the graphs (i.e. approximately ~25 seconds after the start of the measurement).*

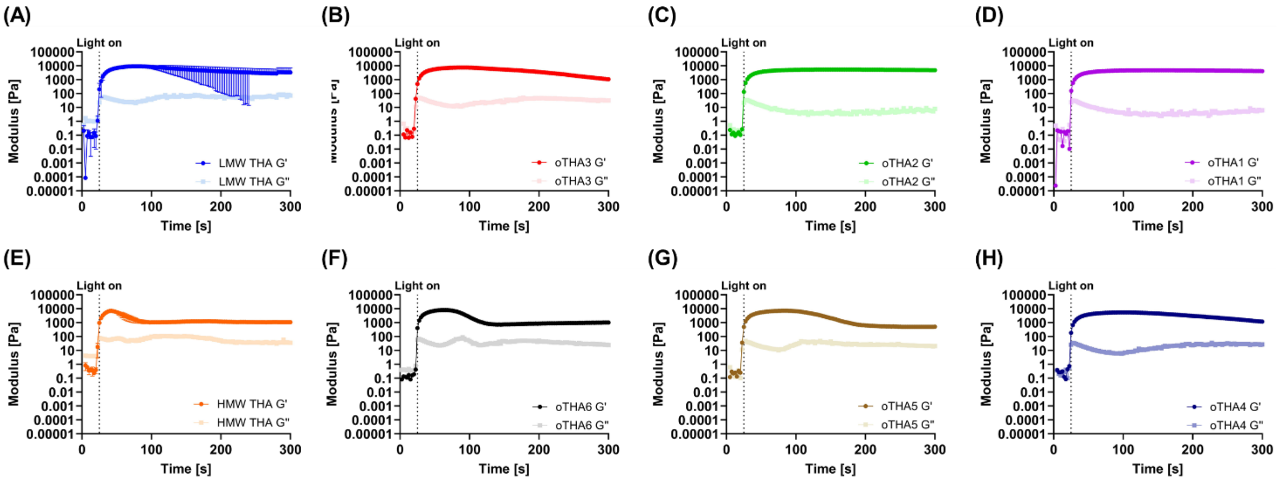

Figure S14. *Gelation kinetics of **(A)** LMW THA, **(B)** oTHA3, **(C)** oTHA2, **(D)** oTHA1, **(E)** HMW THA **(F)** oTHA6, **(G)** oTHA5, **(H)** oTHA4, 2 w/v% hydrogels (with 0.1 mM Ruthenium and 5 mM SPS) from **Set B** of reactions, at 23 °C. Average and standard deviation value is depicted in the graphs with n = 3 or 4 replicates of measurements. In most cases, the standard deviation error bars are smaller than data marks. The in-situ gelation process was measured on the quartz crystal stage by illuminating the bottom of the deposited sample with a full spectrum light as described in methods, and the light was switched on as indicated by the vertical dashed line in the graphs (i.e. approximately ~25 seconds after the start of the measurement).*

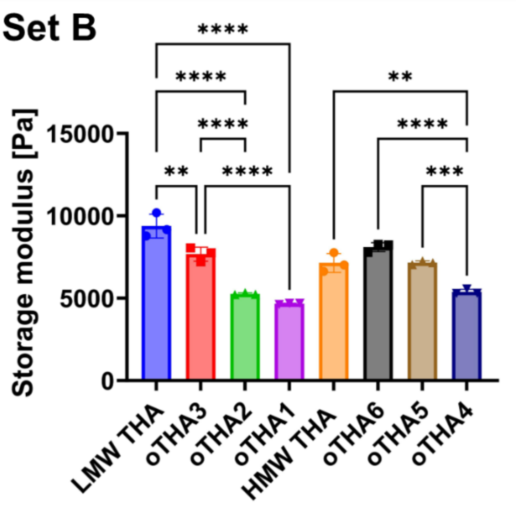

Figure S15. *Storage modulus values obtained for 2 w/v% hydrogels (with 0.1 mM Ru and 5 mM SPS) from Set B, of reactions, extracted as maximum value across the individual measurements depicted in **Figure S14**, at 23 °C. Average and standard deviation value is depicted in the graphs with n = 3 or 4 replicates of measurements. Statistical analysis was done by one-way analysis of variance (ANOVA) with Šídák's multiple comparisons. A statistically significant results were considered for p<0.05 (* - <0.05, ** - <0.01, *** - <0.005 and **** - <0.001).*

The only recorded storage modulus outlier value was for noted for HMW THA in set B (***Figure S15***), which can be due to difference the discrepancy reflecting batch-to-batch variation hydrogel preparation, though procedural inconsistencies during the preparation of these hydrogels from set B.

__Section 4. Supporting data for combining oTHA and THA in a two-component blended hydrogel formulation.__

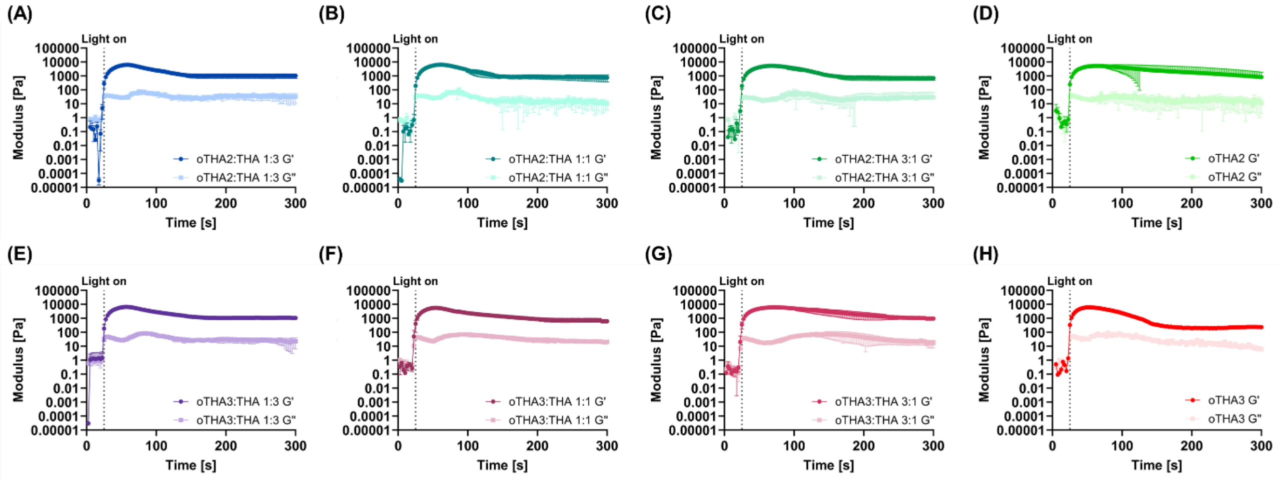

Figure S16. *Gelation kinetics of **(A)** oTHA2:THA 1:3, **(B)** oTHA2:THA 1:1, **(C)** oTHA2:THA 3:1, **(D)** oTHA2, **(E)** oTHA3:THA 1:3 **(F)** oTHA3:THA 1:1, **(G)** oTHA3:THA 3:1, **(H)** oTHA3, 2 w/v% hydrogels (with 0.1 mM Ruthenium and 5 mM SPS) from **Set C** of reactions, at 23 °C. Average and standard deviation value is depicted in the graphs with n = 3 or 4 replicates of measurements. In most cases, the standard deviation error bars are smaller than data marks. The in-situ gelation process was measured on the quartz crystal stage by illuminating the bottom of the deposited sample with a full spectrum light as described in methods, and the light was switched on as indicated by the vertical dashed line in the graphs (i.e. approximately ~25 seconds after the start of the measurement).*

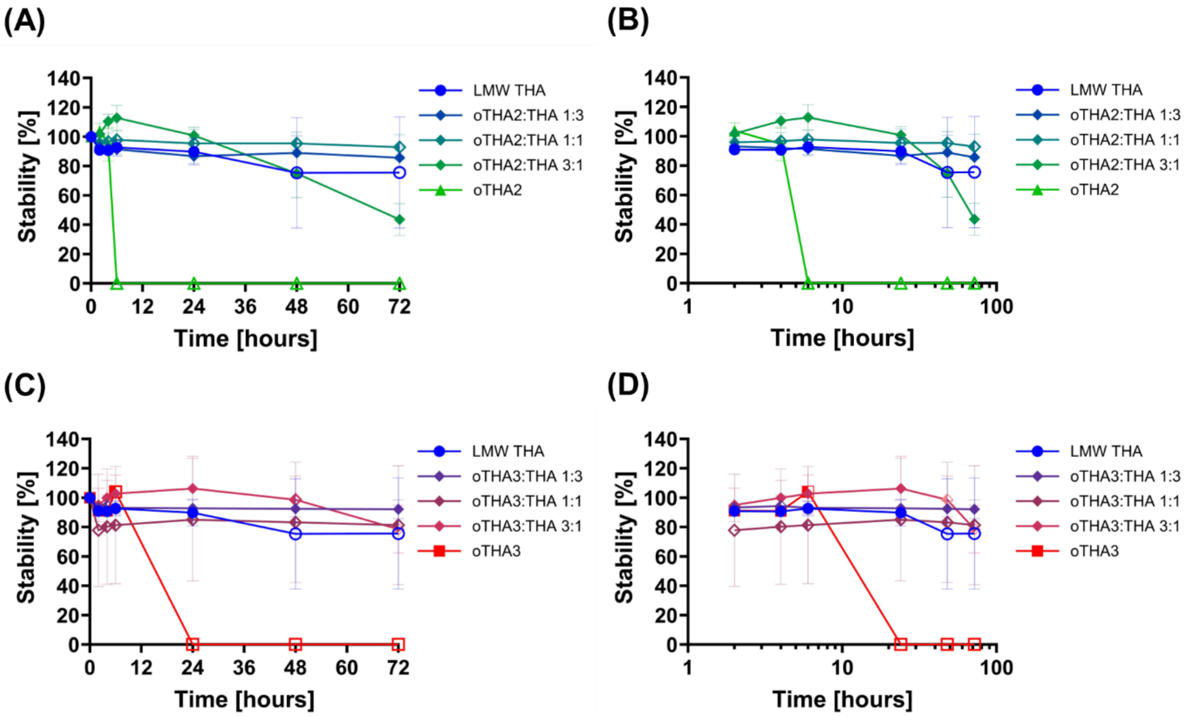

Figure S17. *Swelling profile of two-component oTHA:THA blended hydrogels (2 w/v% total polymer, 0.1 mM Ru, 5 mM SPS) in the absence of hyaluronidase.* ***(A, B)*** *Depicts it for formulations with oTHA2, whereas or* ***(C, D)*** *for oTHA3, as the secondary component.* ***(A)*** *and* ***(C)*** *show standard scale, whereas* ***(B)*** *and* ***(D)*** *are depicted with log10 x-axis scale to better visualize earlier time-points. Average and standard deviation value is depicted in the graphs with n = 5 replicates of all oTHA:THA ratios, n = 4 for all controls, and n = 3 for oTHA2 control.*

Section 5. Supporting data for cartilage ring development and push-out-test adhesion testing.

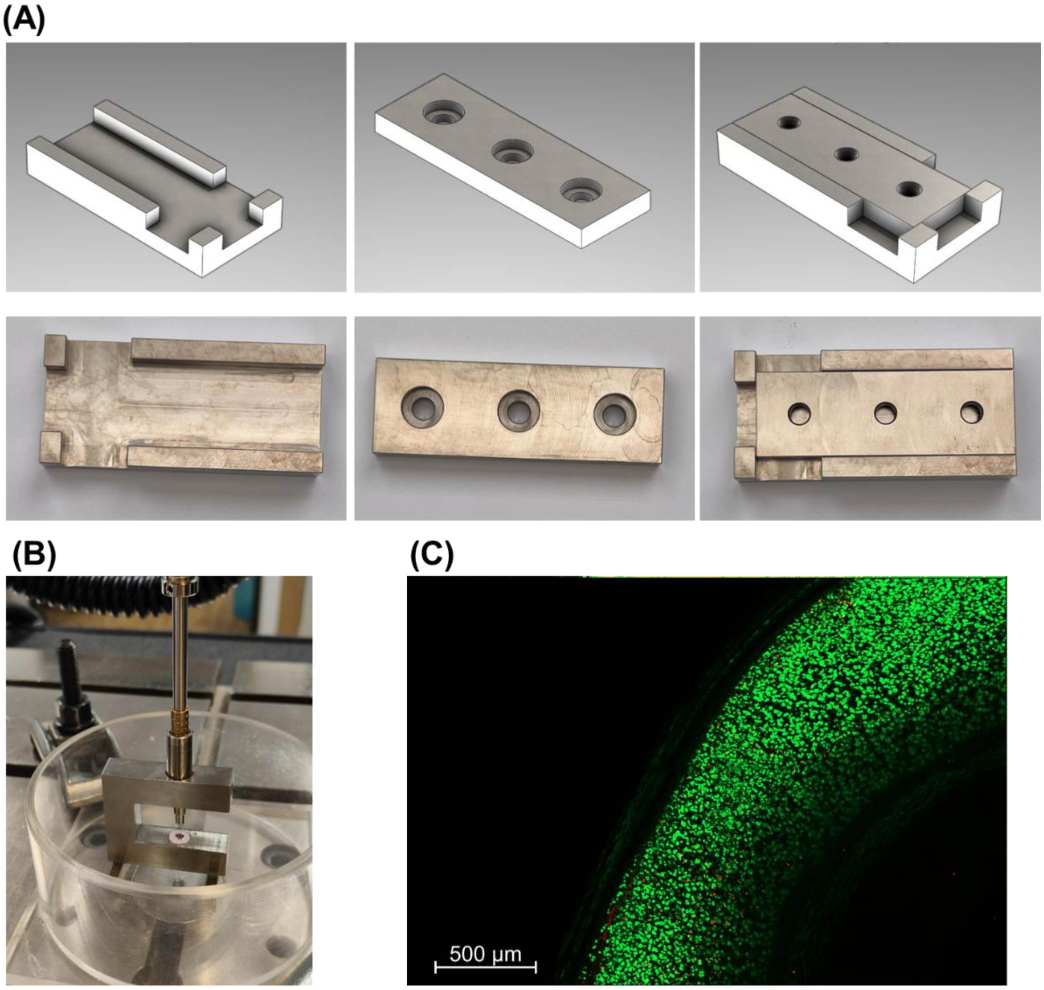

Figure S18. *Workflow for the generation of cartilage rings from the femoral condyles of bovine stifle joints re-adapted from* [9]. ***(A)*** *Newly designed guiding device for generating cartilage rings for the push-out-testing.* ***(B)*** *Image showing setup with the cut cartilage ring placed prior to the measurement.* ***(C)*** *Representative live/dead staining image of as-generated ring at 48-hours of culturing. Full description of this process can be found in methods and in previously published work* [9].

Section 6. Microgels characterization

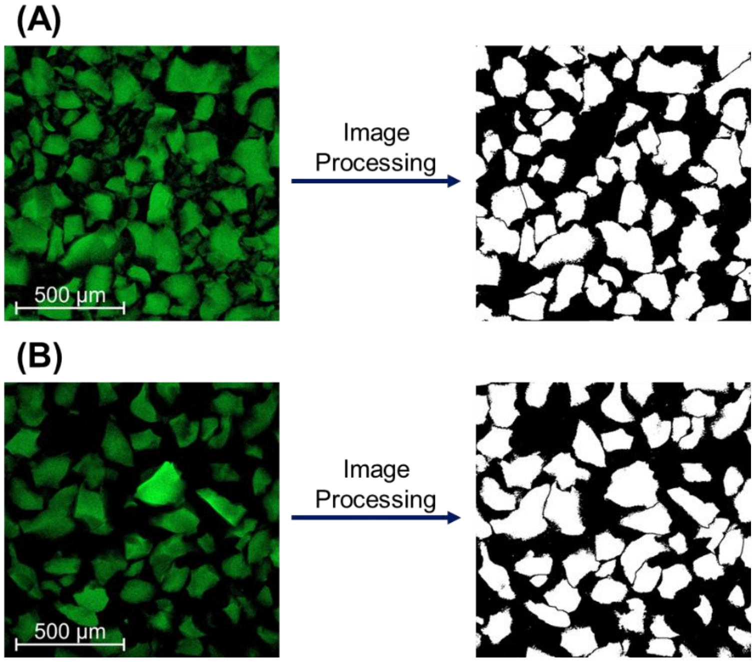

Figure S19. *Microgels obtained from **(A)** oTHA2m and **(B)** oTHA3m from single component hydrogels (2 w/v% total polymer, 0.1 mM Ru, 5 mM SPS), and their workflow for subsequent image analysis. 0.1 wt% of fluorescein isothiocyanate–dextran was included in the hydrogels prior to fragmentation to allow for the imaging.*

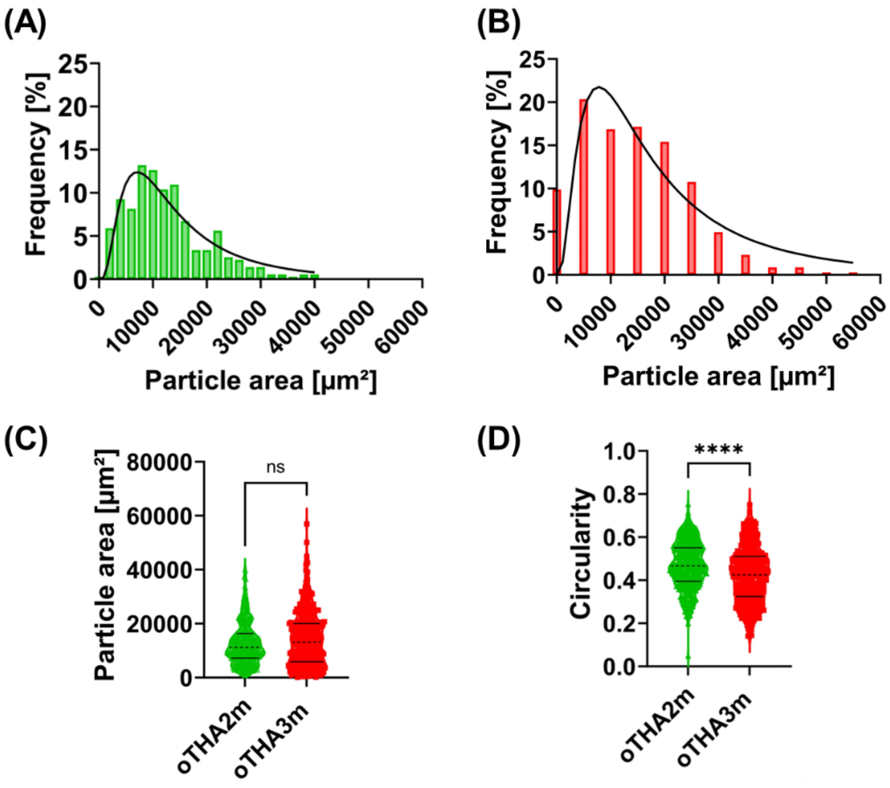

Figure S20. *Area distribution obtained for* ***(A)*** *oTHA2m and* ***(B)*** *oTHA3m microgels (2 w/v% total polymer, 0.1 mM Ru, 5 mM SPS) from n=10 independently taken images.* ***(C)*** *Violin plot of contrasting both sets of microgels.* ***(D)*** *Circularity values of the obtained microgels. Statistical analysis was done by one-way analysis of variance (ANOVA) with Mann-Whitney multiple comparisons; ns = not significant (p>0.05) and significance at p < 0.05 (* < 0.05, ** < 0.01, *** < 0.005, **** < 0.001).*

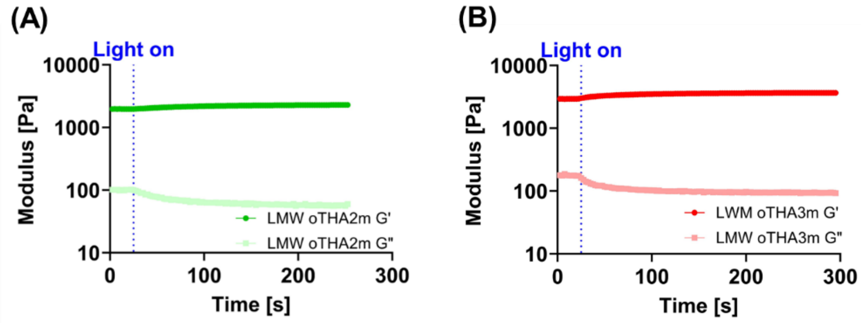

Figure S21. *Gelation kinetics of* ***(A)*** *oTHA2m and* ***(B)*** *oTHA3m, microgels at 2 w/v% hydrogels (with 0.1 mM Ruthenium and 5 mM SPS) from* ***Set C*** *of reactions, at 23 °C. Single repeat is presented here. The in-situ gelation process was measured on the quartz crystal stage by illuminating the bottom of the deposited sample with a full spectrum light as described in methods, and the light was switched on as indicated by the vertical dashed line in the graphs (i.e. approximately ~25 seconds after the start of the measurement). Note: LMW oTHA2m has fewer data points since the activation of the light source was delayed. To synchronize the visualization onset of photo-crosslinking, the 25 s mark was shifted.*

Section 7. Supporting data for combining oTHA microgels within THA matrix – hydrogel microparticles composite approach.

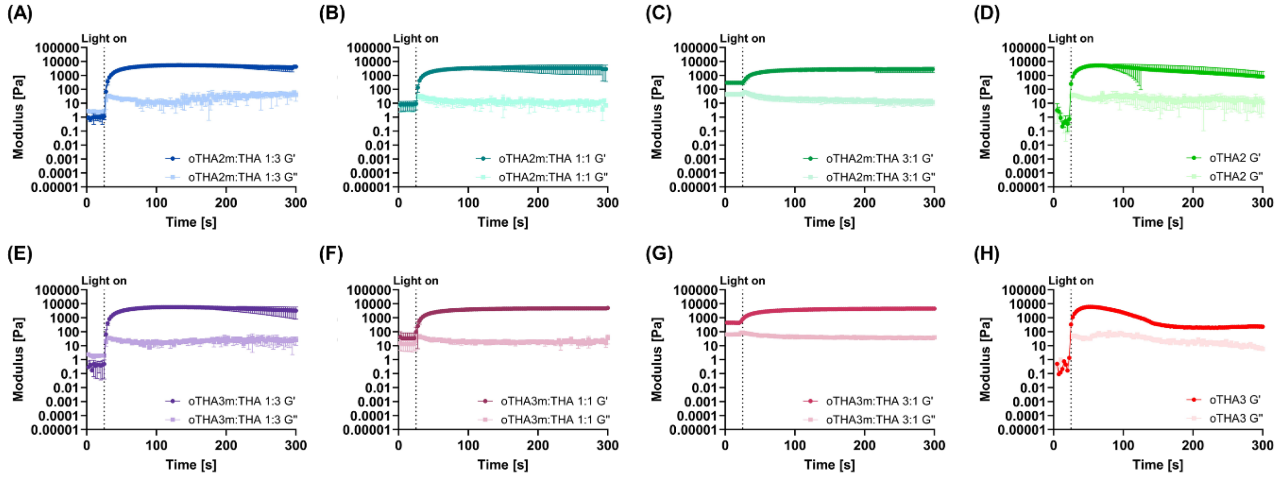

Figure S22. *Gelation kinetics of* ***(A)*** *oTHA2m:THA 1:3,* ***(B)*** *oTHA2m:THA 1:1,* ***(C)*** *oTHA2m:THA 3:1,* ***(D)*** *oTHA2,* ***(E)*** *oTHA3m:THA 1:3* ***(F)*** *oTHA3m:THA 1:1,* ***(G)*** *oTHA3m:THA 3:1,* ***(H)*** *oTHA3, 2 w/v% hydrogels (with 0.1 mM Ruthenium and 5 mM SPS) from* ***Set C*** *of reactions, at 23 °C. Average and standard deviation value is depicted in the graphs with n = 3 or 4 replicates of measurements. In most cases, the standard deviation error bars are smaller than data marks. The in-situ gelation process was measured on the quartz crystal stage by illuminating the bottom of the deposited sample with a full spectrum light as described in methods, and the light was switched on as indicated by the vertical dashed line in the graphs (i.e. approximately ~25 seconds after the start of the measurement).*

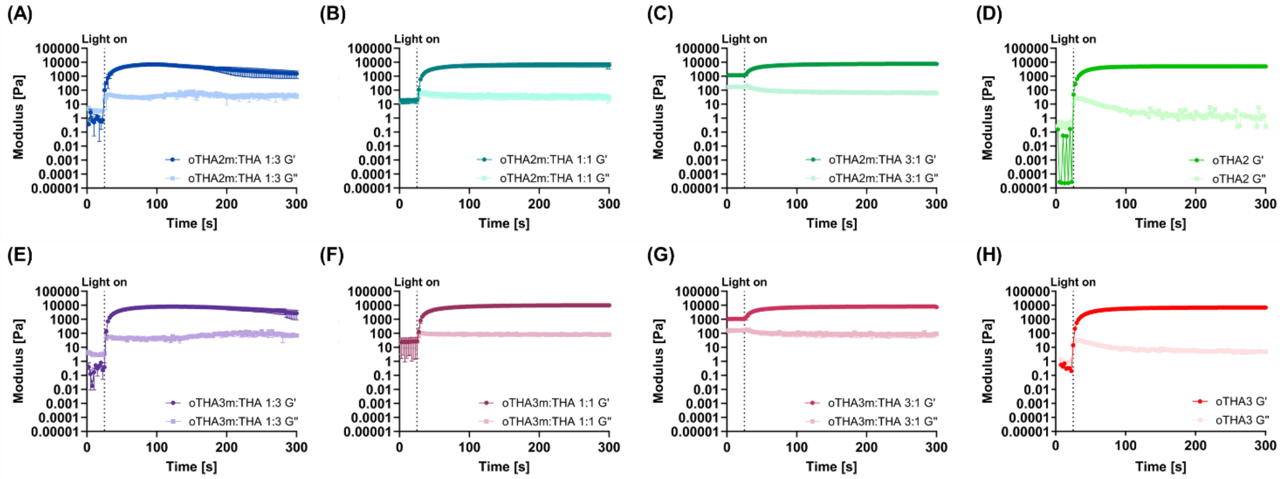

Figure S23. *Gelation kinetics of **(A)** oTHA2m:THA 1:3, **(B)** oTHA2m:THA 1:1, **(C)** oTHA2m:THA 3:1, **(D)** oTHA2, **(E)** oTHA3m:THA 1:3 **(F)** oTHA3m:THA 1:1, **(G)** oTHA3m:THA 3:1, **(H)** oTHA3, 3.5 w/v% hydrogels (with 0.1 mM Ruthenium and 5 mM SPS) from **Set C** of reactions, at 23 °C. For **(A-C)** and **(E-G)**, average and standard deviation value is depicted in the graphs with n = 3 or 4 replicates of measurements. For **(D)** average of n = 2 repeats with standard deviation are depicted, whereas for **(H)** a single measurement is presented. In most cases, the standard deviation error bars are smaller than data marks. The in-situ gelation process was measured on the quartz crystal stage by illuminating the bottom of the deposited sample with a full spectrum light as described in methods, and the light was switched on as indicated by the vertical dashed line in the graphs (i.e. approximately ~25 seconds after the start of the measurement).*

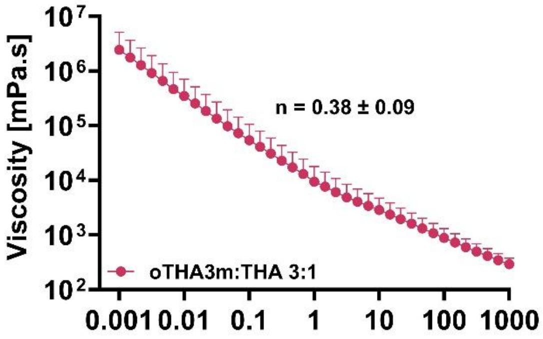

Figure S24. *Steady flow experiments at 23 °C of oTHA3m:THA 3:1 formulation (2 w/v% total polymer, 0.1 mM Ru, 5 mM SPS) with n=2 repeats, and standard deviation noted. Shear exponent (n) was calculated using equation 2 (see methods) and is provided in figure.*

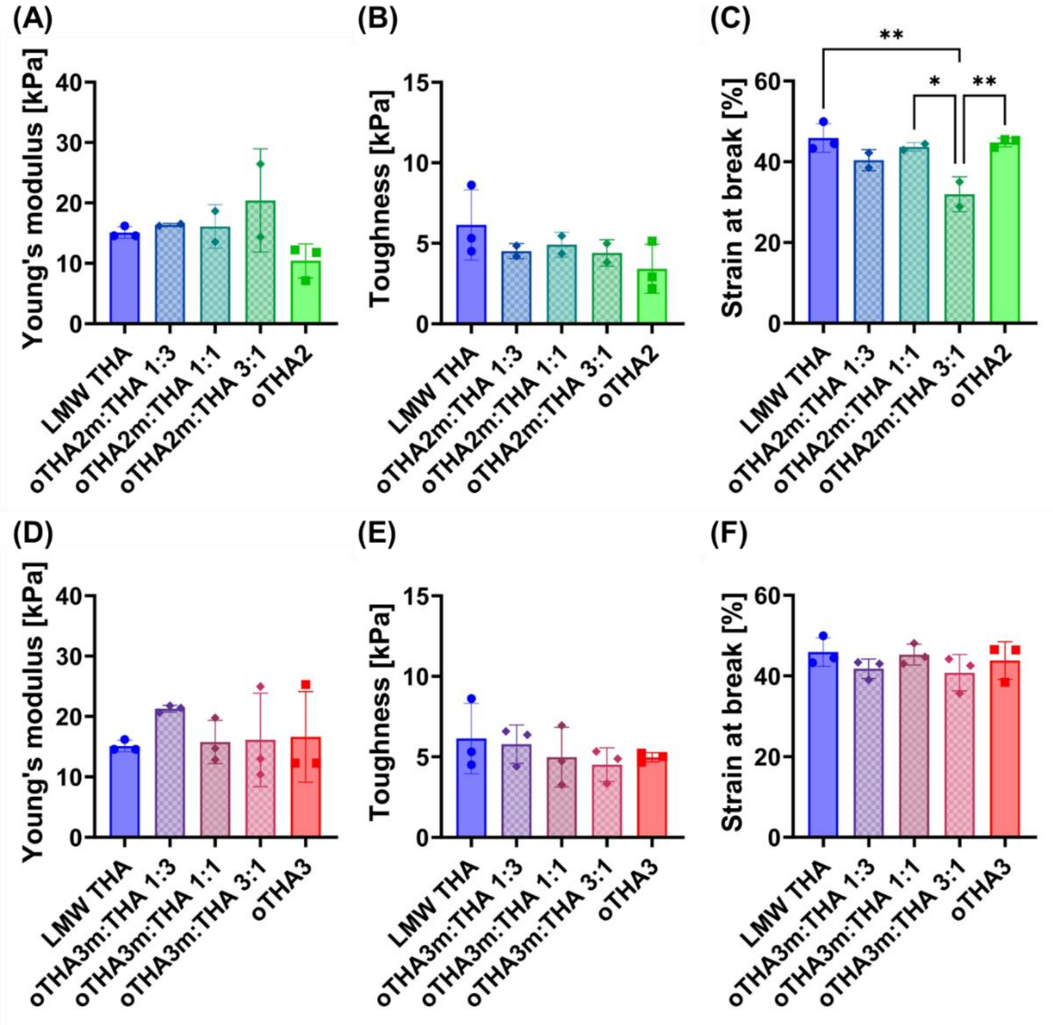

Figure S25. *Compressive testing of hydrogel microparticles composites (2 w/v% total polymer, 0.1 mM Ru, 5 mM SPS).* ***(A)*** *and* ***(D)*** *Young's modulus;* ***(B)*** *and* ***(E)*** *Toughness, and* ***(C)*** *and* ***(F)*** *strain at break, all measured and calculated in compression testing, with n = 3 for controls and oTHA3m samples, and n = 2 for oTHA2m samples. Statistical analysis was done by one-way analysis of variance (ANOVA) with Šídák's multiple comparisons; significance at $p < 0.05$ (* < 0.05, ** < 0.01, *** < 0.005, **** < 0.001).*

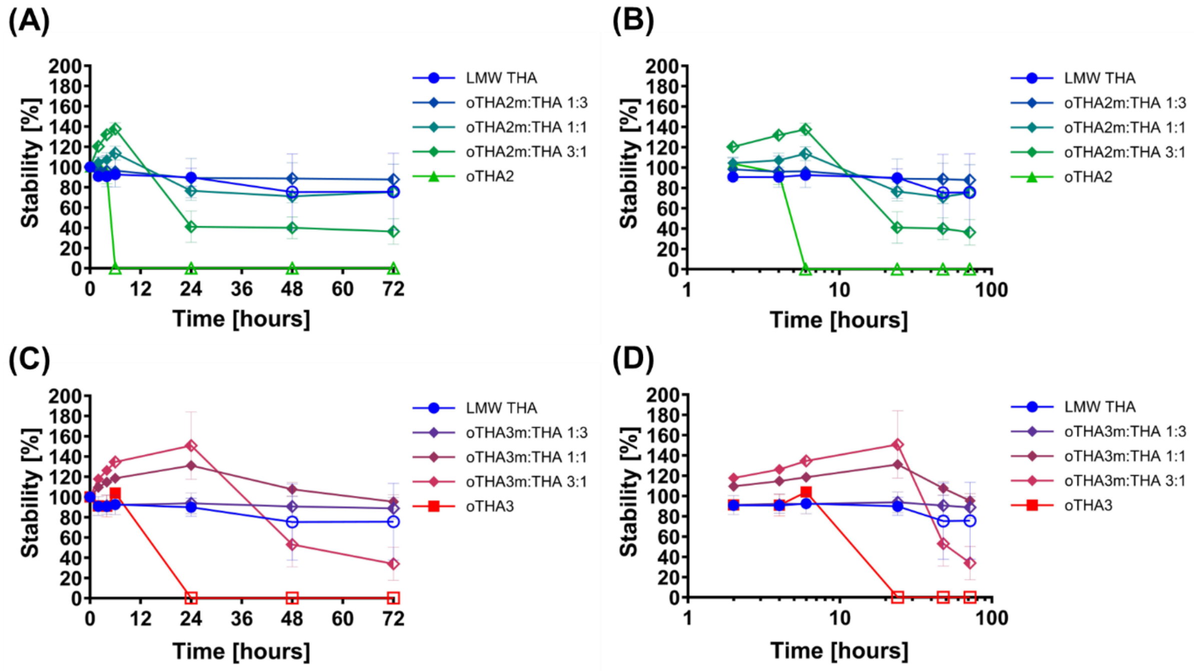

Figure S26. *Swelling profile of oTHAm-THA hydrogel microparticles composites (2 w/v% total polymer, 0.1 mM Ru, 5 mM SPS) in the absence of hyaluronidase.* ***(A, B)*** *Depicts it for formulations with oTHA2m, whereas or* ***(C, D)*** *for oTHA3m, as the secondary component.* ***(A)*** *and* ***(C)*** *show standard scale, whereas* ***(B)*** *and* ***(D)*** *are depicted with log10 x-axis scale to better visualize earlier time-points. Average and standard deviation value is depicted in the graphs with n = 5 replicates of all oTHA:THA ratios, n = 4 for all controls, and n = 3 for oTHA2 control.*

References

(1) Loebel, C.; D'Este, M.; Alini, M.; Zenobi-Wong, M.; Eglin, D. Precise tailoring of tyramine-based hyaluronan hydrogel properties using DMTMM conjugation. *Carbohydrate Polymers* **2015**, *115*, 325-333. DOI: https://doi.org/10.1016/j.carbpol.2014.08.097.

(2) Wychowaniec, J. K.; Bektas, E. I.; Vernengo, A. J.; Muerner, M.; Airoldi, M.; Tipay, P. S.; Sapudom, J.; Teo, J.; Eglin, D.; D'Este, M. Effect of molecular weight of tyramine-modified hyaluronan on polarization state of THP-1 and peripheral blood mononuclear cells-derived macrophages. *Biomaterials Advances* **2025**, *169*, 214166. DOI: https://doi.org/10.1016/j.bioadv.2024.214166.

(3) Chen, H.; Qin, J.; Hu, Y. Efficient Degradation of High-Molecular-Weight Hyaluronic Acid by a Combination of Ultrasound, Hydrogen Peroxide, and Copper Ion. In *Molecules*, 2019; Vol. 24.

(4) Darsy, G.; Patarin, J.; Conrozier, T. Large Variations in Resistance to Degradation between Hyaluronic Acid Viscosupplements: A Comparative Rheological Study. *CARTILAGE* **2023**, *16* (2), 224-231. DOI: 10.1177/19476035231205696

(5) Weis, M.; Shan, J.; Kuhlmann, M.; Jungst, T.; Tessmar, J.; Groll, J. Evaluation of Hydrogels Based on Oxidized Hyaluronic Acid for Bioprinting. In *Gels*, 2018; Vol. 4.

(6) Cowman, M. K.; Schmidt, T. A.; Raghavan, P.; Stecco, A. Viscoelastic Properties of Hyaluronan in Physiological Conditions. *F1000Res* **2015**, *4*, 622. DOI: 10.12688/f1000research.6885.1

(7) Maleki, A.; Kjøniksen, A.-L.; Nyström, B. Effect of pH on the Behavior of Hyaluronic Acid in Dilute and Semidilute Aqueous Solutions. *Macromolecular Symposia* **2008**, *274* (1), 131-140. DOI: https://doi.org/10.1002/masy.200851418

(8) Horkay, F.; Falus, P.; Hecht, A.-M.; Geissler, E. Length Scale Dependence of the Dynamic Properties of Hyaluronic Acid Solutions in the Presence of Salt. *The Journal of Physical Chemistry B* **2010**, *114* (47), 15445-15450. DOI: 10.1021/jp106578f.

(9) Behrendt, P.; Ladner, Y.; Stoddart, M. J.; Lippross, S.; Alini, M.; Eglin, D.; Armiento, A. R. Articular Joint-Simulating Mechanical Load Activates Endogenous TGF-beta in a Highly Cellularized Bioadhesive Hydrogel for Cartilage Repair. *Am J Sports Med* **2020**, *48* (1), 210-221. DOI: 10.1177/0363546519887909
